# Systematic Detection and Identification of Bioactive Ingredients from *Citrus aurantium* L. var. *amara* Using HPLC-Q-TOF-MS Combined with a Screening Method

**DOI:** 10.3390/molecules25020357

**Published:** 2020-01-15

**Authors:** Liuyi Yu, Miaofen Chen, Jinghong Liu, Xiuqiong Huang, Wei He, Zhixing Qing, Jianguo Zeng

**Affiliations:** 1 Hunan Key Laboratory of Traditional Chinese Veterinary Medicine & College of Food Science and Technology, Hunan Agricultural University, Changsha 410128, China; liuyi_yu@yeah.net (L.Y.); dorismfchen@126.com (M.C.); liujinghong1990@126.com (J.L.); huangxqiong@163.com (X.H.); 2 College of Pharmacy, Hunan University of Chinese Medicine, Changsha 410208, China; 3 Green Melody Bio-engineering Group Company Limited, Changsha 410329, China; hewei3218@126.com

**Keywords:** *Citrus aurantium* L. var. *amara*, HPLC-Q-TOF-MS, screening method, metabolites, flavonoids, alkaloids

## Abstract

Bitter orange, *Citrus aurantium* L. var. *amara* (CAVA), is an important crop and its flowers and fruits are widely used in China as a food spice, as well as in traditional Chinese medicine, due to its health-promoting properties. The secondary metabolites that are present in plant-derived foods or medicines are, in part, responsible for the health benefits and desirable flavor profiles. Nevertheless, detailed information about the bioactive ingredients in CAVA is scarce. Therefore, this study was aimed at exploring the phytochemicals of CAVA by high performance liquid chromatography/quadrupole time-of-flight mass spectrometry (HPLC-Q-TOF-MS). Here, a systematic screening method combined with HPLC-Q-TOF-MS was presented. This technique was used to systematically screen metabolites, primarily from the complex matrix of CAVA, and to identify these compounds by their exact masses, characteristic fragment ions, and fragmentation behaviors. A total of 295 metabolites were screened by the screening method and 89 phytochemicals were identified in the flowers, fruits, roots, leaves, and branches of CAVA. For the first time, 69 phytochemicals (flavonoids, alkaloids, terpenoids, etc.) were reported from CAVA. The results highlight the importance of CAVA as a source of secondary metabolites in the food, medicine, and nutraceutical industries.

## 1. Introduction

*Citrus aurantium* L. var. *amara* (CAVA), known as the bitter or sour orange, is a variant of *C. aurantium* L. The flowers and fruits of CAVA are recommended medicine, as well as food, by the Chinese Ministry of Health and are widely distributed in Hunan, Jiangxi, Fujian, Guangdong, and Zhejiang provinces of China [1,2]. They are used for losing weight, reducing sputum, and relieving asthma in traditional Chinese medicine (TCM) [3,4,5]. Recent pharmacological studies have shown that CAVA has potential antioxidant [6], antitumor [7], anti-inflammatory [8], antimicrobial [9], anti-atherosclerosis [10], antianxiety [11], and antiamnesic [12] activities. Flavonoids and alkaloids are regarded as the primary active phytochemicals in CAVA, specifically, flavonoid glycosides, flavone, flavanone, and polymethoxyflavone [13,14,15,16,17]. In addition, CAVA contains volatile oils [18], limonoids [19], and coumarins [19]. In previous studies, only fourteen flavonoids, three coumarins, one limonin, and two alkaloids were purified and isolated from CAVA [3,6,20,21,22]. In addition to these well-known constituents, a number of unknown flavonoids, alkaloids, coumarins, and limonoids were detected by HPLC-Q-TOF-MS in trace amounts and require further investigation.

Systematic phytochemical isolation, bioactivity-guided isolation, and mass spectrometry (MS) guided isolation have all been used, in previous studies, as the three primary methods to isolate and identify unknown compounds [23]. MS-guided isolation is a high efficiency method for detection and isolation of new metabolites or isomers from plant samples. Compared with traditional phytochemical separation methods, this method can avoid repetitive separation of known compounds, and thus has a higher potential for the discovery of new compounds [23,24]. Therefore, it is necessary to identify the chemicals using HPLC-Q-TOF-MS technologies primarily. In this study, we present a comprehensive approach to using HPLC-Q-TOF-MS, combined with a screening strategy as a rapid, sensitive, and simple method for systematic screening and identification of flavonoids, alkaloids, coumarins, and limonoids in CAVA.

In many early studies, the separation and identification of chemical constituents by liquid chromatography mass spectrometry (LC-MS) has been typically performed on a single part of a plant [3,4,21], which caused a number of compounds to be omitted. In this study, samples extracted from the flowers, fruits, leaves, branches, and roots of CAVA were analyzed. Compounds that led to distinct peaks in total ion chromatography (TIC) are easily isolated and identified, but trace components and those exhibiting poor MS response that do not show significant peaks in TIC are difficult to detect and characterize. To solve this problem, a screening protocol, including non-, accurate-, and extensive-target methods was combined to find the flavonoids, alkaloids, coumarins, limonoids, and other compounds in CAVA.

The non-target method is widely used as a traditional and common means to detect compounds by screening the secondary metabolites one-by-one, based on the significant peaks of TIC. However, for some trace components or low MS response compounds, distinct peaks are not formed, and thus are easily missed [24]. The accurate-target method is a means to identify compounds that have been reported in specific plants from previous studies, however, this method is only suited for well-known compounds and does not work for unknown compounds [24]. The extensive-target method is a relatively comprehensive means to screen similar compounds, resulting in the formation of a series of theoretical exact masses by combining known skeletons with common substituent groups in specific plants and, then, screening the theoretical calculated mass from TIC to discover the potential molecular candidates [25]. The above three methods have been used individually for screening metabolites in specific plants, however, systematic detection of compounds by combining the three methods has rarely been reported. In this study, 295 compounds were detected in CAVA by combining all three methods and 89 of them were identified.

## 2. Results and Discussions

### 2.1. Establishment of the Screening Method

**Figure 1:**
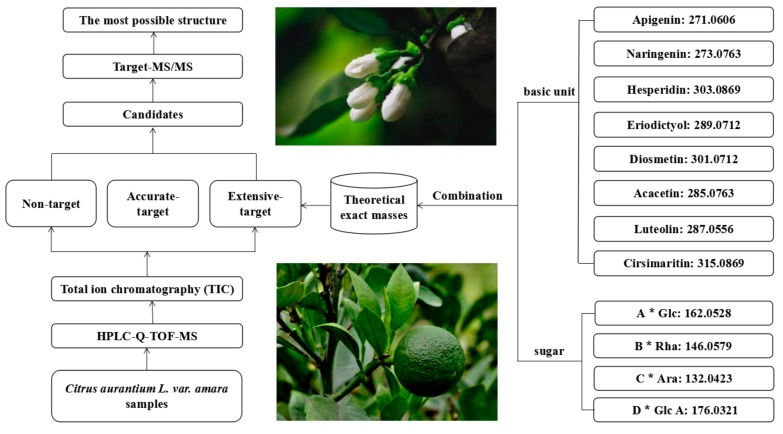
The diagram for systematic screening the secondary metabolites by high performance liquid chromatography/quadrupole time-of-flight mass spectrometry (HPLC-Q-TOF-MS) combined with a screening method. A, B, C, and D represent the number of glucose (Glc), rhamnose (Rha), arabinose (Ara), and glucuronic acid (Glc A), respectively and the number of sugars is no more than 3 according to previous studies.

LC-MS is a fast and sensitive tool for the detection and identification of metabolites in plant medicines and foods. In previous studies, many components, especially trace compounds, have been missed due to poor screening methods. In a specific plant, analogues with the same skeleton but different substituent groups are synthesized synchronously in different amounts through specific biosynthetic pathways. Abundant compounds, or ones exhibiting a high-quality MS response, are easy to detect, while trace analogs, or compounds that exhibit a poor MS response, are always overwhelmed by complex matrices, and are difficult to discover [23,24]. In light of this situation, a method for detecting analogues in CAVA using HPLC-Q-TOF-MS combined with a screening strategy was established (Figure 1). Three approaches, non-, accurate-, and extensive-target were used for systematic screening metabolites from the TICs of different CAVA samples.

A non-target method was used to screen compounds that were abundant or compounds that exhibited a high-quality MS response which can present distinct peaks in TIC and, then, fragment ions were obtained by tandem mass spectrometry (MS/MS). An accurate-target method was performed by first developing a list of all reported compounds in previous studies from the genus including their structure, molecular formula, accurate mass, and identification method. Then, the measured exact masses of candidates were obtained using extracted ion chromatogram (EIC) of the calculated precise mass of reported compounds on the TICs. Finally, the characteristic fragment ions of candidates were produced by target-MS/MS. In this study, 142 previously reported compounds were summarized; 106 components were detected in CAVA using the accurate-target method and 44 of them were identified. An extensive-target method combines known basic molecular units with different sugars to obtain a series of theoretical calculated masses and, then, EIC of the formed theoretical exact masses on the TICs of the samples were performed. If the measured MS data match the theoretical calculated mass, those combined molecules are considered to be present in the sample. Finally, the fragments of each candidates were obtained by target-MS/MS. In this study, 272 theoretical exact masses were formed by combining the eight basic units (hesperitin, naringenin, apigenin, eriodictyol, diosmetin, acacetin, luteolin, and cirsimaritin) with four common sugars (glucose, rhamnose, arabinose, and glucuronic acid). According to the TICs of flowers, fruits, leaves, roots, and branches of CAVA, the measured exact masses of 67 candidates were obtained by using an EIC method, and the target-MS/MS analysis was conducted for each candidate. Finally, the most likely structures of 38 metabolites were inferred by the fragmentation pathway of references. The above three methods have been used individually for screening of metabolites in specific plants, however, comprehensive and systematic detection of bioactive ingredients by combining the three methods has been rarely reported (Figure 1). In this study, 295 compounds were screened from CAVA by combining all three methods and 89 of them were identified.

### 2.2. Screening and Identification of Flavonols and Flavonol Glycosides

A series of similar compounds with the same framework but different substituent groups are distributed throughout CAVA. Since these analogues typically display similar MS fragmentation behaviors, investigation of the fragmentation pathways of well-known references is a valid approach for identifying the unknown analogues. The fragmentation behaviors and characteristic diagnostic ions of ten reference samples were investigated in detail and used for identifying the flavonols and flavonol glycosides in CAVA.

**Figure 2:**
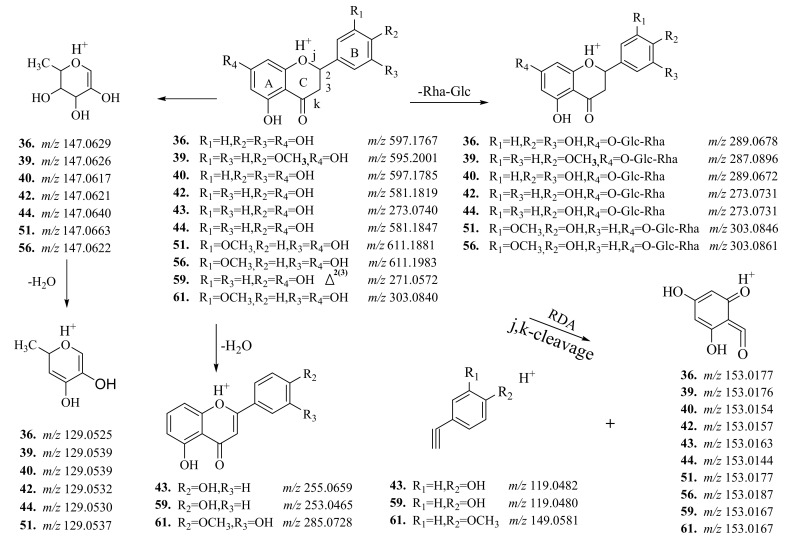
The proposed fragmentation pathways of 10 flavonol and flavonol glycoside references.

In the MS/MS spectra of neoeriocitrin (**36**), poncirin (**39**), eriocitrin (**40**), naringin (**42**), naringenin (**43**), narirutin (**44**), neohesperidin (**51**), hesperidin (**56**), apigenin (**59**), and hesperitin (**61**) (Figure S1), four fragmentation behaviors dominated. The first fragmentation pathway was the successive neutral loss of sugars from the protonated flavonol glycoside, and formation of the basic unit. In the MS/MS spectra of **36**, **39**, **40**, **42**, **44**, **51**, and **56**, the protonated basic unit ions at *m*/*z* 289.0678, 287.0896, 289.0672, 273.0731, 273.0731, 303.0846, and 303.0861 were formed, respectively, by the loss of a Glc-Rha group from the protonated precursor ions at *m*/*z* 597.1767, 595.2001, 597.1785, 581.1819, 581.1847, 611.1881, and 611.1983 (Figure 2). The second fragmentation pattern was the cleavage of the C-ring and formation of a series of relatively low *m*/*z* fragment ions (Figure 2). The characteristic fragment ions at *m*/*z* 153.01, 119.04, and 149.05 for **36**, **39**, **40**, **42**, **43**, **44**, **51**, **56**, **59**, and **61** were produced by a retro Diels–Alder (RDA) reaction (cleavage of j and k-bond of the C-ring). The third fragmentation pattern was the loss of small molecular groups, such as H_2_O and CO, from the basic skeleton and formation of a series of fragment ions. In the MS/MS spectra of compounds **59** and **61**, fragment ions at *m*/*z* 253.0465 and 285.0728 were generated by the loss of H_2_O moiety from the [M + H]^+^ ions with *m*/*z* values of 271.0572 and 303.0840, respectively [26]. The final fragmentation pattern was cleavage of the sugar moiety and formation of a series of relatively low *m*/*z* fragment ions. The characteristic fragment ions at *m*/*z* 147.06 and 129.05 for **36**, **39**, **40**, **42**, **44**, **51** and **56** were formed by cleavage of the sugar moiety. These fragmentation behaviors are considered to be diagnostic pathways for flavonols and flavonol glycosides in CAVA. The proposed fragmentation patterns are shown in Figure 2.

In previous studies, the basic units of flavonols and flavonol glycosides in CAVA were apigenin ([M + H]^+^ *m*/*z* 271.0606), naringenin ([M + H]^+^ *m*/*z* 273.0763), hesperidin ([M + H]^+^ *m*/*z* 303.0869), eriodictyol ([M + H]^+^ *m*/*z* 289.0712), diosmetin ([M + H]^+^ *m*/*z* 301.0712), acacetin ([M + H]^+^ *m*/*z* 285.0763), luteolin ([M + H]^+^ *m*/*z* 287.0556), and cirsimaritin ([M + H]^+^ *m*/*z* 315.0869) [20,21]. The dehydrated glucose (Glc, 162.0528), rhamnose (Rha, 146.0579), arabinose (Ara, 132.0423), and glucuronic acid (Glc A, 176.0321) were the primary substituent groups for those components. By adding no more than three sugar molecules to the skeleton, a total of 272 different theoretical exact masses were obtained. Sixty-seven candidates were produced using EIC based on the TIC of flowers, fruits, roots, leaves, and branches of CAVA and their MS/MS spectra were produced by the target-MS/MS model. The structures of 38 candidates were tentatively determined by the fragmentation pathways of flavonols and flavonol glycosides. In addition, 142 potential compounds were obtained by the non-target and accurate-target methods and 47 compounds were tentatively identified by their characteristic fragmentation behaviors. Finally, 209 flavonols and flavonol glycosides were screened by the non-, accurate-, and extensive-target methods and 58 components, including 19 flavones, 27 flavanones, and 12 polymethoxyflavonoids were identified and 45 of them were reported for the first time from this plant.

**Figure 3:**
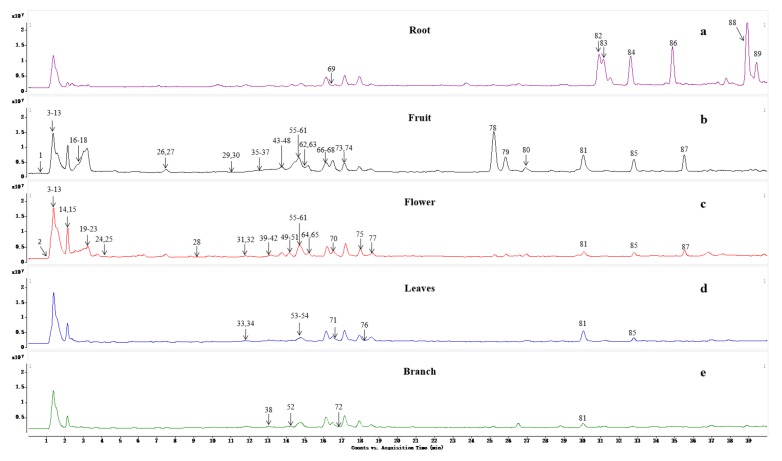
The total ion chromatogram (TIC) of root (**a**), fruit (**b**), flower (**c**), leaves (**d**), and branch (**e**) of *Citrus aurantium* L. var. *amara* (CAVA).

**Figure 4:**
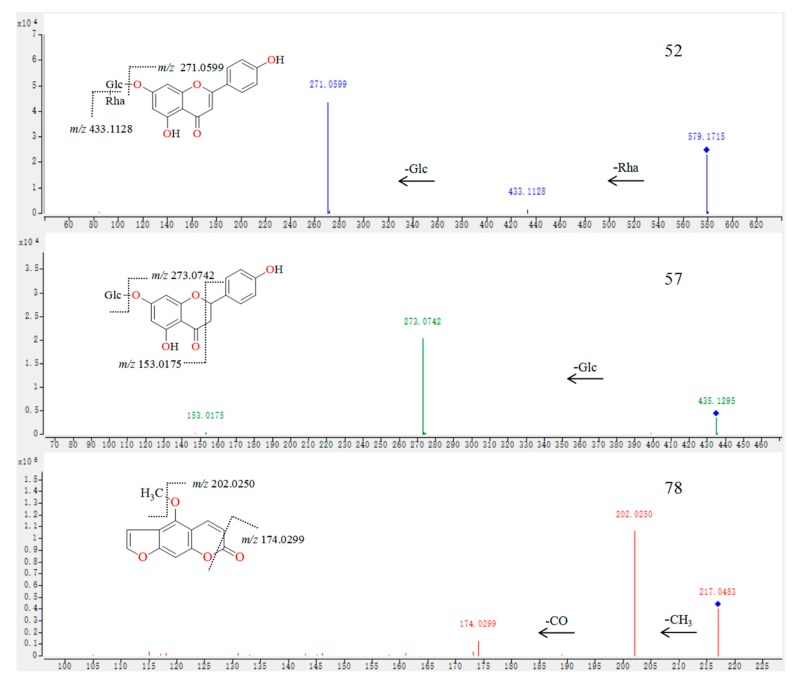
The tandem mass spectrometry (MS/MS) spectra and fragmentation behaviors of compounds **52**, **57**, and **78**.

Compound **59** was screened by the three screening methods simultaneously and its MS/MS data was obtained by target-MS/MS. Compound **59** was identified as apigenin unambiguously by comparison of the retention time, MS, and MS/MS data with that of the standard (Table 1). It was difficult to find compound **52** (TR = 14.22 min, Figure 3) using the non-target method because of the low content or poor response and the lack of distinct peaks in the TICs. However, this compound was easily detected by the accurate- and extensive-target methods using EIC on the TIC of different parts of CAVA. In the MS/MS spectrum of compound **52** (Figure 4), the fragment ion occurring at *m*/*z* 433.1128 was observed for the loss of a Rha residue from the protonated ion at *m*/*z* 579.1715. Subsequently, the absence of the Glc moiety was found and formed the basic skeleton at *m*/*z* 271.0599, indicating the presence of the -Glc-Rha group in the structure of compound **52**. The characteristic ions occurring at *m*/*z* 271.0599, 153.0153, and 129.0525 demonstrated that the basic skeleton for compound **52** was apigenin. Therefore, compound **52** was tentatively identified as apigenin-*O*-glucoside-*O*-rhamnoside (Figure 4). Using the same method, the remaining flavone-type compounds (**28**, **29**, **30**, **31**, **32**, **38**, **45**, **50**, **55**, **58**, **62**, **63**, **65**, **66**, **67**, **70**, and **73**) were provisionally identified (Table 1) and the relevant MS/MS spectra are provided in the Supplementary Materials (Figure S2). 

Compounds **36**, **39**, **40**, **42**, **43**, **44**, **51**, **56**, and **61** were unambiguously identified as neoeriocitrin, poncirin, eriocitrin, naringin, naringenin, narirutin, neohesperidin, hesperidin, and hesperitin, respectively, by comparison of the retention time, MS, and MS/MS data with the corresponding standards (Table 1). The protonated ion of compound **57** was submerged in high abundance ions or complex biological matrices, making it difficult to detect by the non-target method (Figure 3). In addition, this secondary metabolite has not been reported in this genus previously. Therefore, not surprisingly, it was difficult to detect compound **57** by the accurate-target mean, however, the compound was detected with the extensive-target method by screening the theoretical exact mass on the TICs. The extensive-target method indicated that the basic skeleton of this compound was naringenin and the substituent group was Glc. In the MS/MS spectrum of compound **57** (Figure 4), the basic skeleton ion at *m*/*z* 273.0742 was formed by the loss of a Glc residue from the protonated ion occurring at *m*/*z* 435.1295. The fragments at *m*/*z* 153.0175 and 273.0742 indicated that the basic skeleton was naringenin. Thus, compound **57** was preliminarily identified as naringenin-*O*-glucoside (Figure 4). Using the same method, the remaining flavanone-type compounds (**33**, **34**, **35**, **37**, **41**, **46**, **47**, **48**, **49**, **53**, **60**, **64**, **68**, **69**, **74**, **75**, and **77**) were tentatively identified (Table 1) and the relevant MS/MS spectra are provided in the Supplementary Materials (Figure S2).

**Figure 5:**
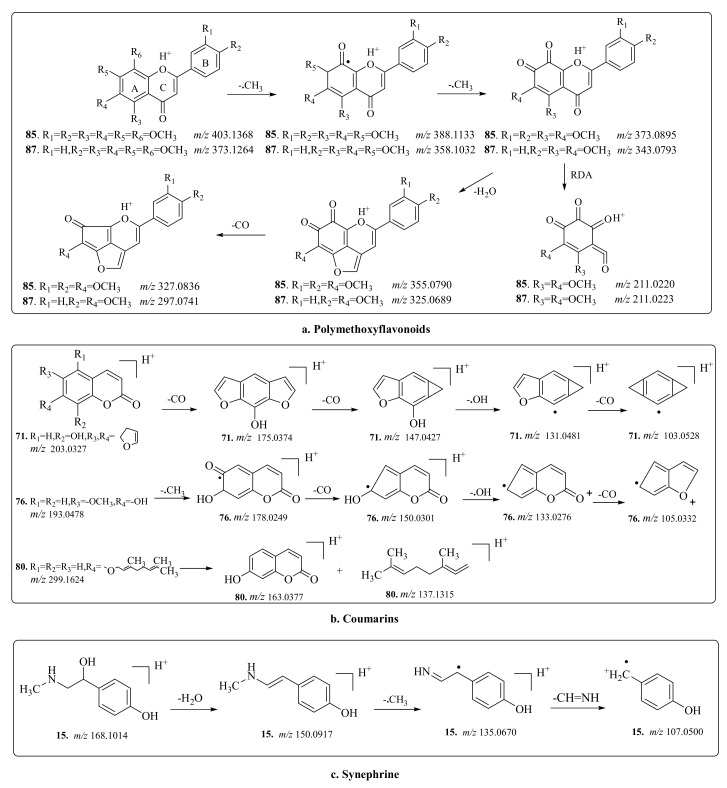
The proposed fragmentation pathways of six references. (**a**) Polymethoxyflavonoids; (**b**) Coumarins; (**c**) Synephrine.

Compounds **85** and **87** were unambiguously identified as nobiletin and tangeretin, respectively, by comparing the retention time, MS, and MS/MS data with the references (Table 1). Compound **86** presented a distinct peak in the TIC of CAVA roots (Figure 3) and has previously been reported in this genus. Therefore, compound **86** was easily detected with the non-target and accurate-target methods. The fragmentation pathways of polymethoxyflavonoid-type compounds were investigated in detail using nobiletin (**85**) and tangeretin (**87**) as references before identifying their structures of compound **86** and other compounds. The MS behaviors of polymethoxyflavonoid-type compounds were different from other types of flavonoids. First, this type of compound only responded well in positive mode of ESI. Second, the main fragmentation route was the successive losses of small groups, such as H_2_O moiety and CH_3_ radical from the basic skeleton. In the MS/MS spectra of references **85** and **87** (Figure S1), fragment ions observed at *m*/*z* 388.1133 and 358.1032 were generated by loss of CH_3_ radical from the protonated ions at *m*/*z* 403.1368 and 373.1264, respectively (Figure 5a). Fragments at *m*/*z* 355.0790 and 325.0689 were formed by neutral loss of H_2_O moiety from the ions at *m*/*z* 373.0895 and 343.0793, respectively. The third fragmentation pattern was cleavage of the C-ring and formation of relatively low *m*/*z* fragment ions. The characteristic fragment ions at *m*/*z* 211.0220 and 211.0223 for compounds **85** and **87** were formed by RDA reaction (cleavage the C-ring, Figure 5). In the MS/MS spectrum of compound **86**, the fragmentation behavior had a high similarity with polymethoxyflavonoid-type compounds. The difference in *m*/*z* values of compounds **86** and **87** was 30.0079 Da, which indicated that the structure of compound **86** has an OCH_3_ moiety fewer than that of compound **87**. According to a previous report [27], compound **86** was preliminarily identified as 4′,5,6,7-pentamethoxyflavone by comparison with characteristic ions. Using the similar method, the remaining polymethoxyflavonoid-type compounds (**26**, **54**, **72**, **81**, **82**, **83**, **84**, **88**, and **89**) were tentatively identified (Table 1) and the relevant MS/MS spectra are provided in the Supplementary Materials (Figure S2).

### 2.3. Screening and Identification of Coumarin

In the MS/MS spectra of the three references (xanthotoxol (**71**), scopoletin (**76**), and auraptene (**80**) (Figure S1), it is difficult to cleave the skeleton of coumarin, therefore, the primary characteristic fragmentation pathway was the loss of small molecular groups, such as CO, CH_3_, and OH, from the basic skeleton. In the MS/MS spectra of compounds **71** and **76**, fragment ions at *m*/*z* 175.0347, 147.0427, 103.0528, 150.0301, and 105.0332 were generated by the loss of a CO moiety from the protonated ions at *m*/*z* 203.0327, 175.0374, 131.0481, 178.0249, and 133.0276, respectively. In the MS/MS spectra of compounds **71** and **76**, fragment ions at *m*/*z* 131.0481 and 133.0276 were produced by the loss of a OH radical from the protonated ions at *m*/*z* 147.0427 and 150.0301, respectively. The proposed characteristic fragmentation pathways are shown in Figure 5b.

Compounds **71**, **76**, and **80** were clearly identified as xanthotoxol, scopoletin, and auraptene (Table 1), respectively, by comparing the retention time, MS, and MS/MS data with those of the standards. Compound **78** presented a distinct peak in the TICs (Figure 3). In addition, this compound has been previously reported in this genus. Therefore, compound **80** could be detected easily by the non- and accurate-target methods. In the MS/MS spectrum of compound **78**, the fragmentation behavior was highly consistent with coumarin-type compounds. The difference in *m*/*z* values between compounds **78 and 71** was 14.0146 Da, which indicated that compound **78** has a CH_3_ moiety more than compound **71**. In the MS/MS spectrum of compound **78**, the high abundance fragment ion at *m*/*z* 202.0250 was generated by the loss of a CH_3_ radical from the protonated ion at *m*/*z* 217.0483, which indicated that a CH_3_ moiety was included in the structure of metabolite **78**. According to a previous report [28], compound **78** was preliminarily identified as bergapten (Figure 4).

### 2.4. Screening and Identification of Alkaloids and Triterpenoid

In the MS/MS spectrum of synephrine (**15**) (Figure S1), the primary fragmentation route was the loss of small molecular groups, such as CH_3_ and H_2_O, from the basic skeleton. The fragment observed at *m*/*z* 150.0917 was formed by the neutral loss of H_2_O from the protonated ion at *m*/*z* 168.1014. Successive loss of CH_3_ and CHNH moieties were observed, resulting in the ions at *m*/*z* 135.0670 and 107.0500. The proposed fragmentation pathways are shown in Figure 5c.

By comparing the retention time, MS, and MS/MS data with the standard substance, the structure of compound **15** was clearly determined (Table S1). Compound **21** presented a distinct peak in the TICs (Figure 3) and has been reported previously in this genus. Therefore, compound **21** was detected easily by the non- and accurate-target methods. In the MS/MS spectrum of compound **21**, the difference in *m*/*z* value between compounds **21** and **15** was 15.9908 Da, which indicates that the structure of compound **21** results from the loss of an OH moiety in compound **15**. Moreover, the MS/MS fragmentation behaviors of both compounds are highly similar. The ion observed at *m*/*z* 121.0639 was generated corresponds to the loss of -NH_2_CH_3_ from the protonated ion at *m*/*z* 152.1065. The subsequent loss of an H_2_O moiety and formation of a peak at *m*/*z* 103.0528 was also observed. The MS/MS data indicates that -NHCH_3_ and -OH groups are present in the structure of compound **21**. According to the previous report [29], it was preliminarily identified as *N*-acetylnorsynephrine. Using the same method, the remaining alkaloids (**2**, **7**, **10**, **11**, **14**, **16**, **17**, **18**, **19**, **20**, **24**, **25**, and **27**) were tentatively identified (Table 1) and the relevant MS/MS spectra are provided in the Supplementary Materials (Figure S2).

Limonin compounds have been reported previously as the most common triterpenoids in CAVA [20]. Compound **79** was screened by non-target and accurate-target methods and its structure was unambiguously identified as limonin by comparing the retention time, MS and MS/MS data with the reference (Table 1).

### 2.5. Distribution of Metabolites in CAVA

The distribution of all identified compounds in roots, fruits, flowers, leaves, and branches of CAVA were determined using EIC, based on the TICs. More than 90% ingredients were detected and identified from the flowers and fruits, however, the number of identified metabolites from other parts were relatively small. This is the reason why the flowers and fruits were used as main medicinal parts in traditional Chinese medicine. Flavonoids, alkaloids, and coumarins were the main active ingredients of the flowers and fruits. Thirty-two characteristic compounds, such as limonin (**79**) and auraptene (**80**), were detected only from both parts. Flavonoids and flavonoid glycosides were the main metabolites of flowers, however, the polymethoxyflavonoid-type compounds (such as **78**, **81, 85,** and **87**) were only in fruits, which demonstrated that the flowers and fruits have different functions as herbal medicine or food additives. The polymethoxyflavonoid-type compounds were detected only from the family of *Citrus reticulata* Blanco in previous studies and have a wide range of biological activities, such as antioxidant, anti-inflammatory, antitumor, and antifungal activity [3,30]. Those types of flavonoids were mainly distributed in the fruits and roots of CAVA. Nevertheless, the species and amounts of polymethoxyflavonoid-type compounds have a huge difference between the two parts. Some high content polymethoxyflavonoids (such as compounds **82**, **83, 88,** and **89**, comparing the relative peak area) were detected only from the roots of CAVA. Although compounds **84** and **86** were found in fruits and roots, the level of both compounds in roots was far more than that in fruits. Interestingly, some high content polymethoxyflavonoids (such as compounds **78, 79, 81**, and **85**) in fruits were difficult to detection in the roots (Figure 3). The results revealed that the roots of CAVA, usually discarded in the previous disposal process, were an important source of polymethoxyflavonoid-type metabolites for the food, medicine, and nutraceutical industries.

## 3. Conclusions

In this study, we have shown that the combination of HPLC-Q-TOF-MS with a systematic screening method constitutes a powerful analytical tool for the detection and identification of bioactive ingredients in all parts of CAVA. A total of 295 secondary metabolites were primarily found from the flowers, fruits, roots, leaves, and branches of CAVA with a systematic screening method, which is comprised of non-, accurate-, and extensive-target approaches. Eighty-nine compounds, including 19 flavones, 27 flavanones, 12 polymethoxyflavonoids, 4 coumarins, 15 alkaloids, 1 limonoids, and 11 other phytochemicals were identified by their exact masses, fragment ions, and characteristic fragmentation patterns. Sixty-nine of the compounds are reported for the first time from CAVA. To the best of our knowledge, this work marks the first comprehensive study of secondary metabolites from different parts of CAVA. In addition, the established screening method can also be applied to other plant-derived foods and medicines for systematic detection of the bioactive compounds from the complex biological matrices.

## 4. Materials and Methods

### 4.1. Materials and Chemicals

Deionized water was used for HPLC-Q-TOF-MS and HPLC-Q-TOF-MS/MS analysis and was purified using a Milli-Q system (Merck Millipore, Billerica, MA, USA). HPLC-grade acetonitrile and formic acid were purchased from Merck (Darmstadt, Germany) and ROE (Newark, New Castle, USA), respectively. Methanol (AR) was purchased from the National Institutes for Food and Drug Control (Beijing, China). The seventeen reference substances (seven flavonol glycosides, three flavonols, two polymethoxyflavonoids, three coumarins, one alkaloid, and one limonin), including synephrine (**15**), neoeriocitrin (**36**), poncirin (**39**), eriocitrin (**40**), naringin (**42**), naringenin (**43**), narirutin (**44**), neohesperidin (**51**), hesperidin (**56**), apigenin (**59**), hesperitin (**61**), xanthotoxol (**71**), scopoletin (**76**), auraptene (**80**), limonin (**79**), nobiletin (**85**), and tangeretin (**87**) were purchased from Shanghai Yuanye Bio-Technology Co., Ltd. (Shanghai, China).

### 4.2. Sample Collection and Preparation

The flowers, fruits, roots, branches, and leaves of CAVA were collected from LianYuan KangLu biotechnology company (Hunan, China, GPS coordinates are E 111° 51′ 23.95′ and N 27° 49′ 33.52′) and were authenticated by Prof Jianguo Zeng (Hunan Agricultural University, China). All plant parts of CAVA were freeze-dried and a portion of each was crushed using a disintegrator. Powdered samples (~0.1 g) were extracted with methanol aqueous solution (10 mL, 80% *v*/*v*) using an ultrasonic bath for 45 min. Samples were filtered through a 0.22 μm nylon membrane before injection onto the HPLC-Q-TOF-MS system.

### 4.3. HPLC-Q-TOF-MS Conditions

Agilent 1290 HPLC system (Agilent Technology, Santa Clara, California, USA) was used for the chromatography, consisting of a rapid resolution binary pump, auto-sampler, thermostated column compartment, vacuum degasser, and tunable UV detector. Separation was carried out on a Unitary-C18 column (150 mm × 2.1 mm, 2.8 μm, Accrom Co., Ltd., Dilian, China). The elution system was 0.1% aqueous formic acid solution (A) and acetonitrile (B), while linear gradient elution optimization was performed as follows: 0 to 10 min, 5% to 20% (B); 10 to 30 min, 20% to 50% (B); and 30 to 40 min, 50% to 90% (B). The flow rate was set at 0.3 mL/min, the column temperature was kept at 35 ℃, and the sample injection volume was 5 μL.

MS spectrometry experiment was performed using a 6530 Q-TOF/MS accurate-mass spectrometer (Agilent Technology) in positive electrospray ionization mode. Time-of-flight data were collected between *m*/*z* 100 to 1700 in centroid mode. The conditions for Q-TOF-MS optimization were as follows: Gas temperature, 345 ℃; fragmentor voltage, 150 V; sheath gas flow rate, 11 L/min; sheath gas temperature, 350 ℃; nebulizer gas pressure, 50 psi; sheath gas flow, 11 L/min; capillary voltage, 4000 V; OCT1 RF Vpp, 750 V; and skimmer voltage, 65 V. The TOF MS spectrometer was continuously calibrated, and *m*/*z* 121.050873 and 922.009798 were used as reference solution masses to obtain high-precision mass measurement results. The targeted MS/MS experiments were conducted with variable collision energy (10 to 105 eV) to optimize each compound.

## Figures and Tables

**Table 1:** The peak number (PN), retention time (TR), MS^1^, molecular formula, characteristic fragment ions, identification, screening method, and metabolic distribution of the screened and identified target compounds.

PN	T_R_ (min)	MS^1^ ^b^ (ppm)	Formula	Characteristic MS/MS Ions (*m*/*z*)	Identification	Screening Method	Plant Part
1 ^b^	0.67	163.1116 (0.8)	C_11_H_14_O	117.0720, 89.0605, 57.0691	Citrus H	X	Flower, root, fruit, leaf, branch
2 ^b^	1.08	130.0862 (0.4)	C_6_H_11_NO_2_	84.0784, 70.0643	Pipecolic acid	X, Y	Flower, root, fruit, leaf, branch
3 ^b^	1.21	133.0600 (5.8)	C_4_H_8_N_2_O_3_	116.0335, 87.0543, 74.0230	Asparagine	Y	Flower, root, fruit, leaf, branch
4 ^b^	1.23	277.1270 (4.2)	C_12_H_20_O_7_	259.0916, 211.0695, 133.0596	Citrus I	X	Flower, root, fruit, leaf, branch
5 ^b^	1.24	147.0764 (0.1)	C_5_H_10_N_2_O_3_	130.0494, 101.0701, 84.0436	Glutamine	Y	Flower, fruit, leaf, branch
6 ^b^	1.26	106.0500 (−1.2)	C_3_H_7_NO_3_	88.0389, 70.0286, 60.0437	Citrus J	X	Flower, fruit, leaf, branch
7 ^b^	1.29	156.0397 (4.3)	C_5_H_5_N_3_O_3_	110.0712, 83.0606	Citrus A	X	Flower, root, fruit, leaf, branch
8 ^b^	1.32	175.1193 (−2.0)	C_6_H_15_N_4_O_2_	158.0929, 130.0962, 116.0698, 70.0643, 60.0548	Arginine	Y	Flower, fruit, leaf
9 ^b^	1.38	247.1933 (−6.4)	C_14_H_22_N_4_	144.0948, 58.0704	Citrus K	X	Flower, fruit, leaf
10 ^b^	1.46	248.1993 (6.4)	C_16_H_25_NO	202.0961, 104.1008	Citrus B	X	Flower, root, fruit, leaf, branch
11 ^b^	1.52	314.1586 (3.8)	C_15_H_23_NO_6_	152.1000, 121.0561, 91.0456	*N*-acetylnorsynephrine-rhamnoside	X	Flower, root, fruit, leaf, branch
12 ^b^	1.53	104.1067 (2.8)	C_5_H_13_NO	60.0807, 58.0649	Citrus M	X	Flower, fruit, leaf
13 ^b^	1.58	116.0711 (−4.3)	C_5_H_10_NO_2_	86.0162, 70.0649	Citrus N	X	Flower, fruit, leaf
14 ^b^	1.93	170.1214 (−9.2)	C_9_H_16_NO_2_	152.0893, 124.0686, 97.0715, 91.0471	Dihydro-synephrine	X	Flower, fruit
15 ^a^	2.11	168.0973 (8.4)	C_9_H_14_NO_2_	150.0874, 135.0633, 119.0453, 91.0507	Synephrine	X, Y	Flower, fruit
16 ^b^	2.56	300.1432 (3.2)	C_14_H_22_NO_6_	282.1317, 138.0897, 121.0647	Citrus C	X	Flower, fruit, leaf, branch
17 ^b^	2.60	268.1039 (0.5)	C_10_H_14_N_5_O_4_	136.0605, 119.0328	Adenosine	X, Y	Flower, fruit, leaf, branch
18 ^b^	2.61	284.0973 (5.8)	C_10_H_14_N_5_O_5_	267.1405, 152.0561, 135.0262	Hydroxyadenosine	X	Flower, fruit, leaf, branch
19 ^b^	3.34	222.1120 (2.1)	C_12_H_15_NO_3_	205.1423, 87.0435	Citrus D	X	Flower, fruit
20 ^b^	3.35	240.1032 (−5.4)	C_15_H_13_NO_2_	222.1059, 208.0996, 195.0853, 149.0818, 121.0559, 105.0280	Citrus E	X	Flower, fruit
21	3.37	152.1065(3.2)	C_9_H_13_NO	121.1639, 103.0538, 77.0378	*N*-acetylnorsynephrine	X, Y	Flower, fruit
22 ^b^	3.38	367.1830 (−6.8)	C_25_H_22_N_2_O	322.1387, 229.1012, 58.0654	Citrus L	X	Flower, fruit
23 ^b^	3.41	163.0384 (3.5)	C_9_H_6_O_3_	89.0585, 57.0698	Citrus O	X	Flower, fruit
24 ^b^	3.76	120.0808(−0.2)	C_8_H_9_N	103.0533, 91.0532, 77.0381	Citrus F	X	Flower, fruit
25 ^b^	3.78	166.0856(3.9)	C_9_H_12_NO_2_	131.0488, 120.0799, 103.0535	Phenylalanine	X, Y	Flower, fruit
26 ^b^	7.55	463.1550 (8.2)	C_23_H_26_O_10_	313.0728, 185.0925, 153.0174	3′,4′,5′-trimethoxyflavone-*O*-arabinoside	X	Fruit
27 ^b^	7.59	180.1013 (3.3)	C_10_H_13_NO_2_	163.1293, 107.0683, 89.0588	Citrus G	X	Flower, fruit
28	9.18	595.1657 (0.1)	C_27_H_30_O_15_	449.1104, 287.0537, 147.0639	Luteolin-*O*-glucoside-*O*-rhamnoside	Y, Z	Flower, fruit
29 ^b^	10.86	627.1564(−1.3)	C_27_H_30_O_17_	465.0947, 303.0482, 145.0516	3′,4′,5′,5-hydroxy-flavone-*O*-glucoside-*O*-glucoside	X	Flower, fruit
30 ^b^	11.17	447.1252 (7.5)	C_22_H_22_O_10_	285.0702, 121.0957	Acacetin-*O*-glucoside	Z	Flower, fruit
31 ^b^	11.74	449.1104 (−5.7)	C_21_H_20_O_11_	287.0521, 147.0512, 129.0525	Luteolin-*O*-glucoside	X, Y, Z	Flower, fruit
32 ^b^	11.75	611.1636 (−4.8)	C_27_H_30_O_16_	449.1117, 287.0532	Luteolin-*O*-glucoside-*O*-glucoside	Y, Z	Flower, fruit
33 ^b^	11.79	451.1244 (−2.0)	C_21_H_22_O_11_	289.0704, 153.0186, 107.0429	Eriodictyol-*O*-glucoside	Y, Z	Leaf, branch
34 ^a^	11.88	289.0700 (2.3)	C_15_H_12_O_6_	163.0365, 153.0167, 145.0281	Eriodictyol	X, Y, Z	Leaf, branch
35 ^b^	12.25	595.2077 (−8.4)	C_28_H_34_O_14_	449.1455, 303.0851, 153.0231	Hesperitin-*O*-rhamnoside-*O*-rhamnoside	Z	Flower, fruit
36 ^a^	12.30	597.1835 (−3.5)	C_27_H_32_O_15_	451.1220, 289.0698, 153.0161, 147.0624, 129.0518	Neoeriocitrin	X, Y, Z	Flower, fruit
37 ^b^	12.31	597.1861 (−9.8)	C_27_H_32_O_15_	449.1391, 287.0606	4′-hydroxyl-flavanone-*O*-glucoside -*O*-rhamnoside	X	Flower, fruit
38 ^b^	13.11	463.1244 (−1.9)	C_22_H_22_O_11_	377.9540, 301.0700, 121.1001	Luteolin-*O*-glucoside	X, Y, Z	Flower, fruit, leaf, branch
39 ^ab^	13.20	595.2018 (0.5)	C_28_H_34_O_14_	449.1436, 287.0897, 129.0540	Poncirin	X, Y	Flower, fruit, root
40 ^a^	13.27	597.1818(1.2)	C_27_H_32_O_15_	331.07, 9289.0709, 147.0259	Eriocitrin	Y, Z	Flower, fruit, root
41 ^b^	13.29	419.1186 (−0.4)	C_17_H_22_O_12_	273.0757, 153.0152, 129.0556	Naringenin-*O*-rhamnoside	X, Y, Z	Flower, fruit, root
42 ^a^	13.39	581.1818 (8.0)	C_27_H_32_O_14_	435.1291, 273.0727, 153.0179, 147.0641, 129.0526	Naringin	Y, Z	Flower, fruit, root
43 ^a^	13.49	273.0808 (3.0)	C_8_H_16_O_10_	153.0187, 147.0390, 119.0456	Naringenin	X, Y, Z	Flower, fruit
44 ^ab^	13.67	581.1841 (4.1)	C_27_H_32_O_14_	419.1302, 273.0723, 147.0623, 129.0532	Narirutin	X, Y, Z	Flower, fruit
45	13.69	609.1805 (1.4)	C_28_H_32_O_13_	463.1229, 301.0701, 129.0644, 85.0277	Diosmetin-*O*-glucoside-*O*-rhamnoside	X, Y, Z	Flower, fruit, leaf, branch
46 ^b^	13.76	419.1391(−11.6)	C_21_H_22_O_9_	383.1107,285.0716,129.0534	4′-Methoxy-flavanone-*O*-arabinose	X, Y	Flower, fruit
47 ^b^	13.82	727.2451 (−0.9)	C_33_H_42_O_18_	527.1484, 419.1294, 315.0925129.0538	4′,5′-Methoxy-flavanone-*O*-rhamnoside-*O*-arabinose-*O*-arabinose	X	Flower, fruit
48 ^b^	13.98	565.1954 (−6.7)	C_16_H_12_O_5_	419.1351, 285.0881, 147.0503	4′-Methoxy-flavanone-*O*-rhamnoside-*O*-arabinoside	X	Flower, fruit
49 ^b^	14.15	449.1458 (−3.5)	C_22_H_24_O_10_	413.1228, 303.0833	Hesperitin-*O*-rhamnoside	Y, Z	Flower, fruit, Root, leaf, branch
50 ^b^	14.17	593.1495 (1.0)	C_28_H_32_O_14_	447.1265, 285.0711	Acacetin-*O*-glucuronic acid-*O*-arabinoside	Z	Flower, fruit, Leaf, branch
51 ^a^	14.19	611.1937 (5.4)	C_28_H_34_O_15_	449.1412, 303.0835, 129.0524	Neohesperidin	X, Y, Z	Flower, fruit, Leaf, branch
52 ^b^	14.22	579.1715 (−1.5)	C_27_H_30_O_14_	271.0599, 153.0153, 129.0525	Apigenin-*O*-glucoside-*O*-rhamnoside	Y,Z	Flower, fruit, Root, leaf, branch
53 ^b^	14.23	449.1441 (0.2)	C_22_H_24_O_10_	303.0855, 153.0199,129.0541	Hesperitin-*O*-rhamnoside	X, Z	Flower, fruit, Root, leaf, branch
54 ^b^	14.44	345.0951 (5.1)	C_18_H_16_O_7_	303.0801, 195.0277, 153.0201	3′,4′,5′-trimethoxyflavone	Y	Leaf, branch
55 ^b^	14.57	593.1861 (6.5)	C_28_H_32_O_14_	447.1259, 315.0863, 153.0152	Cirsimaritin-*O*-arabinose	Z	Flower, fruit, branch
56 ^a^	14.58	611.1961 (1.5)	C_28_H_34_O_15_	449.1416, 303.0826, 129.0536	Hesperidin	X, Y, Z	Flower, fruit, root, leaf, branch
57 ^b^	14.61	435.1295 (−2.1)	C_21_H_22_O_10_	273.0742, 153.0175	Naringenin-*O*-glucoside	Z	Flower, fruit, root
58 ^b^	14.65	757.2226 (−5.3)	C_39_H_50_O_25_	611.1763, 287.0477, 129.0537	Luteolin-*O*-glucoside-*O*-rhamnoside-*O*-glucoside	X, Z	Flower, fruit, branch
59 ^ab^	14.68	271.0581 (6.7)	C_15_H_10_O_5_	243.0623, 153.0167, 119.0479	Apigenin	X, Y, Z	Flower, fruit, branch
60 ^b^	14.70	757.2214 (−3.7)	C_33_H_40_O_20_	449.1448, 303.0853, 129.0528	Hesperitin-*O*-glucuronic acid-*O*-arabinoside-*O*-rhamnoside	Z	Flower, fruit, Root, leaf, branch
61 ^a^	14.71	303.0850 (4.3)	C_16_H_14_O_4_	177.0538, 153.0365, 145.0269	Hesperitin	X, Y, Z	Flower, fruit, root
62 ^b^	14.86	739.2450 (−0.8)	C_34_H_42_O_18_	575.1642, 413.1240, 315.0863	Cirsimaritin-*O*-arabinoside-*O*-rhamnoside-*O*-rhamnoside	Z	Flower, fruit, branch
63 ^b^	15.17	653.1725 (−1.9)	C_29_H_32_O_17_	347.0759, 129.0522	3′,3,5-hydroxy-4′,5′-Methoxy-flavone-*O*-glucoside-*O*-rhamnoside	X	Flower, fruit
64 ^b^	15.23	435.1251 (8.0)	C_20_H_18_O_11_	273.0730, 153.0193, 147.0478	Naringenin-*O*-glucoside	X, Z	Flower, fruit
65 ^b^	15.23	609.1797 (2.7)	C_28_H_32_O_15_	301.0690, 463.1244, 153.0151	Diosmetin-*O*-glucoside-*O*-rhamnoside	X, Y, Z	Flower, fruit
66 ^b^	15.96	579.1723 (−2.5)	C_22_H_22_O_10_	433.1139, 271.0590, 129.0534	Apigenin-*O*-glucoside-*O*-rhamnoside	Z	Fruit
67	16.12	463.1213 (4.7)	C_22_H_22_O_11_	445.0233, 301.0707	Diosmetin-*O*-glucoside	X, Y, Z	Flower, fruit, Root, leaf, branch
68	16.16	465.1389 (0.5)	C_22_H_24_O_11_	345.1045, 303.0839, 153.0100	Hesperitin-*O*-glucoside	Y, Z	Flower, fruit
69 ^b^	16.36	667.2219 (2.0)	C_31_H_38_O_16_	521.1088, 273.0693	Naringenin-*O*-arabinoside-*O*-rhamnoside-*O*-arabinoside	Z	Leaf, root branch
70 ^b^	16.52	725.2224 (9.1)	C_33_H_40_O_18_	461.1186, 315.0884, 129.0551	Cirsimaritin-*O*-rhamnoside-*O*-arabinoside-*O*-arabinoside	X, Z	Flower, fruit, Root, leaf, branch
71 ^a^	16.83	203.0337 (0.9)	C_11_H_6_O_4_	175.0382, 147.0439, 119.0479	Xanthotoxol	X, Y	Flower, root, Fruit, leaf, branch
72 ^b^	16.86	491.1511 (7.5)	C_24_H_26_O_11_	345.0871, 153.0143	3′,4′,5′-trimethoxyflavone-*O*-rhamnoside	X	Root, branch
73 ^b^	17.12	755.2379 (1.8)	C_34_H_42_O_19_	597.1865, 271.0806, 127.0386	Apigenin-*O*-glucuronic acid-*O*-arabinoside-*O*-rhamnoside	Z	Flower, fruit, root, leaf, branch
74 ^b^	17.37	465.1427 (−6.5)	C_22_H_24_O_11_	303.0861, 153.0165	Hesperitin-*O*-glucoside	Y, Z	Flower, fruit, Root, leaf, branch
75 ^b^	17.88	579.1971 (8.3)	C_28_H_34_O_10_	301.1401, 245.0759, 153.0158	4′-Methoxy-flavanone-*O*-rhamnoside-*O*-arabinoside	X, Y	Flower, fruit, Root, leaf, branch
76 ^a^	18.24	193.0476 (7.8)	C_10_H_8_O_4_	178.0247, 150.0302, 133.0275	Scopoletin	X, Y	Flower, root, fruit, leaf, branch
77 ^b^	18.72	755.2382 (1.4)	C_34_H_42_O_19_	609.1823, 303.0831, 153.0207	Hesperitin-*O*-glucosideacid-*O*-rhamnoside-*O*-rhamnoside	Z	Flower, fruit
78 ^b^	25.46	217.0483 (5.7)	C_12_H_9_O_4_	202.0250, 174.0299, 161.0581146.0345, 131.0486, 115.0532	Bergapten	X, Y	Flower, fruit
79 ^a^	25.83	471.2025 (−2.4)	C_26_H_31_O_8_	425.1931, 161.0614	Limonin	X, Y	Flower, fruit
80 ^a^	26.55	299.1620 (7.2)	C_19_H_22_O_3_	163.0375, 137.1314	Auraptene	X, Y	Flower, fruit
81 ^b^	29.95	343.1168 (2.3)	C_19_H_18_O_6_	302.1577, 296.8757	3′,4′,6,7-tetramethoxyflavone	Y	Fruit
82 ^b^	31.21	389.1226 (1.2)	C_20_H_20_O_8_	374.1078, 369.0821, 107.9603	Hydroxy-4′,5′,6,7,8-pentamethoxyflavone	X	Root
83 ^b^	31.57	375.1050 (6.5)	C_19_H_18_O_8_	303.1685	5,7-hydroxy-3′,4′,5′,6-tetramethoxyflavone	X	Root
84 ^b^	32.60	373.1280 (0.4)	C_20_H_20_O_7_	358.0954, 343.0845	4′,5,6,7,8-pentamethoxy-flavone	X, Y	Flower, fruit, root
85 ^a^	32.77	403.1389 (−0.3)	C_21_H_22_O_8_	388.1152, 373.0920, 355.070702, 327.0810,301.0723	Nobiletin	X, Y	Flower, fruit, leaf, branch
86 ^b^	35.26	343.1176 (0.0)	C_19_H_18_O_6_	328.0955, 313.0692, 285.0704	4′,5,6,7-tetramethoxyflavone	X, Y	Flower, fruit, root
87 ^a^	35.48	373.1255 (6.7)	C_20_H_20_O_7_	358.1044, 343.0802, 328.07013250701	Tangeretin	X, Y	Flower, fruit, Leaf, branch
88 ^b^	38.87	373.1288 (−1.6)	C_20_H_20_O	358.1046, 343.0811, 325.0664	4′,5′,6,7,8-pentamethoxy-flavone	X	Root
89 ^b^	39.41	389.1222 (2.3)	C_20_H_20_O_8_	371.2897, 374.1026, 359.0627, 356.0858	5-hydroxy-3′,4′,6,7,8-pentamethoxyflavone	X	Root

^a^, those compounds were unambiguously identified by comparing the retention time, MS, and characteristic MS/MS ions with standards. ^b^, those compounds were reported for the first time in CAVA. X, those compounds were screened by the non-target method. Y, those compounds were screened by the accurate-target method. Z, those compounds were screened by the extensive-target method.

## Supplementary Materials

The Supplementary Materials are available online.
