# Supplementary material for: Systematic Detection and Identification of Bioactive Ingredients from Citrus aurantium L. var. amara Using HPLC-Q-TOF-MS Combined with a Screening Method
Source: Molecules. 2020 Jan 15;25(2):357. doi: 10.3390/molecules25020357 (PMC7024317; doi:10.3390/molecules25020357)
Supplement: Supplementary file 1 [file molecules-25-00357-s001.pdf]

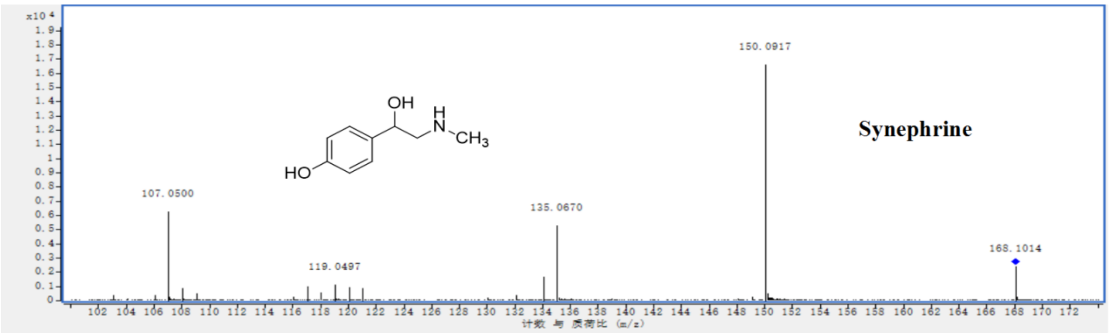
Synephrine
x10 4
107. 0500
119. 0497
135. 0670
150. 0917
168. 1014
OH
H
N
CH3
HO
计数 与 质荷比 (m/z)

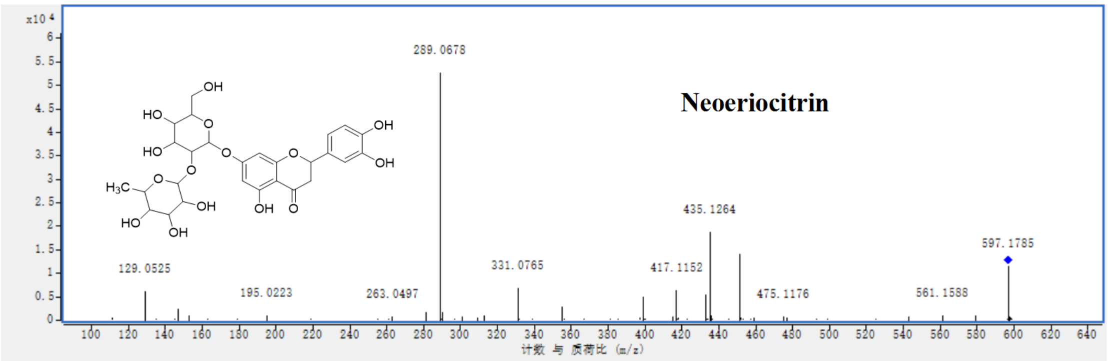
Neoeriocitrin
x10 4
129. 0525
195. 0223
263. 0497
289. 0678
331. 0765
417. 1152
435. 1264
475. 1176
561. 1588
597. 1785
计数 与 质荷比 (m/z)

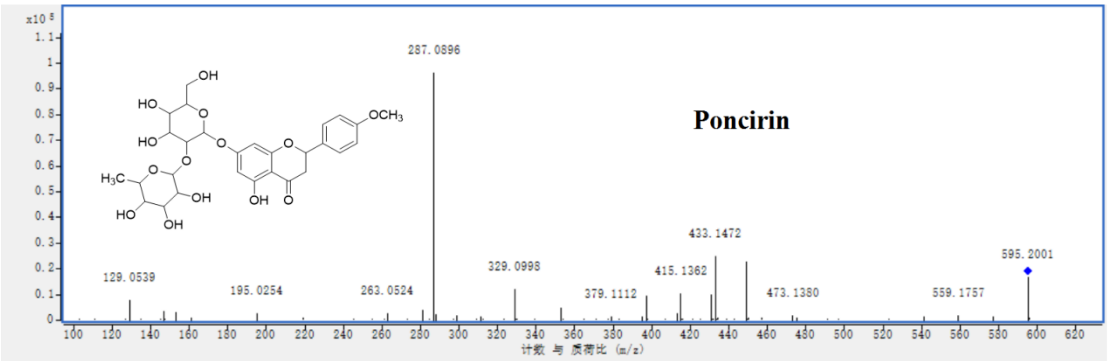
Poncirin
x10 5
129. 0539
195. 0254
263. 0524
287. 0896
329. 0998
379. 1112
415. 1362
433. 1472
473. 1380
559. 1757
595. 2001
OCH3
计数 与 质荷比 (m/z)

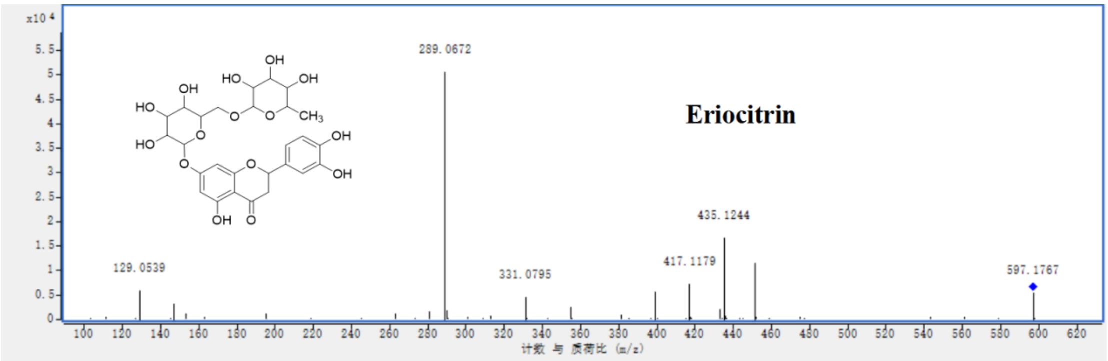
Eriocitrin
x10 4
129. 0539
289. 0672
331. 0795
417. 1179
435. 1244
597. 1767
CH3
计数 与 质荷比 (m/z)

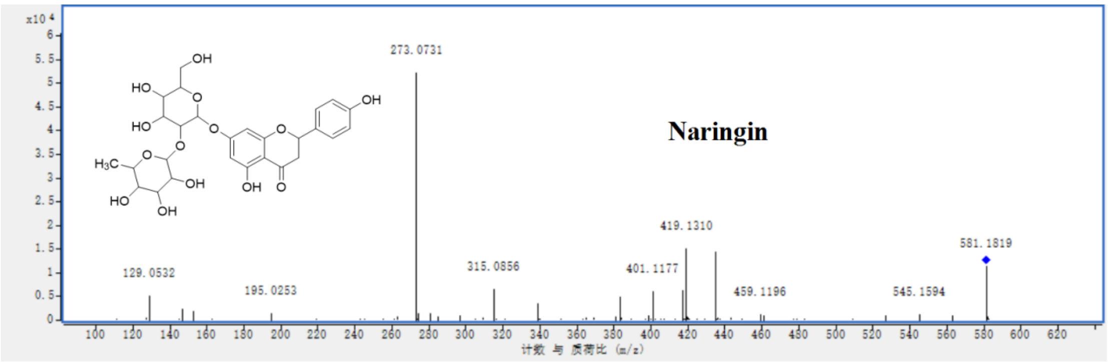
Naringin
x10 4
129. 0532
195. 0253
273. 0731
315. 0856
401. 1177
419. 1310
459. 1196
545. 1594
581. 1819
计数 与 质荷比 (m/z)

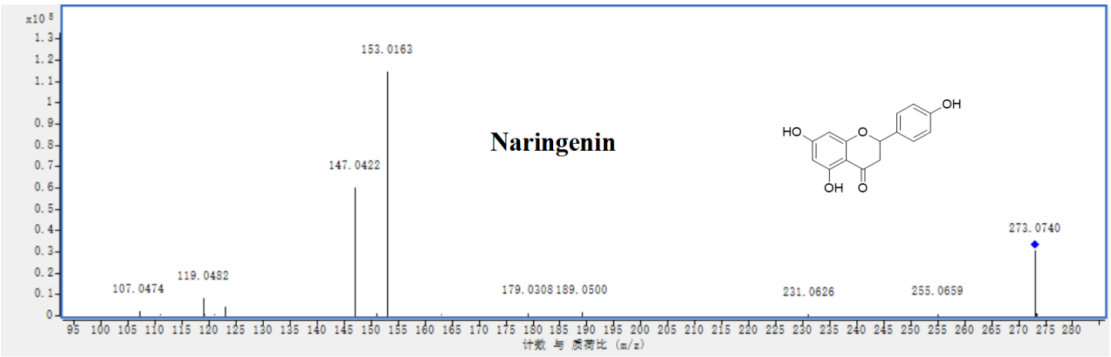

Naringenin
x10 5
153. 0163
147. 0422
119. 0482
107. 0474
179. 0308
189. 0500
231. 0626
255. 0659
273. 0740
HO
OH
OH
O
O
计数 与 质荷比 (m/z)

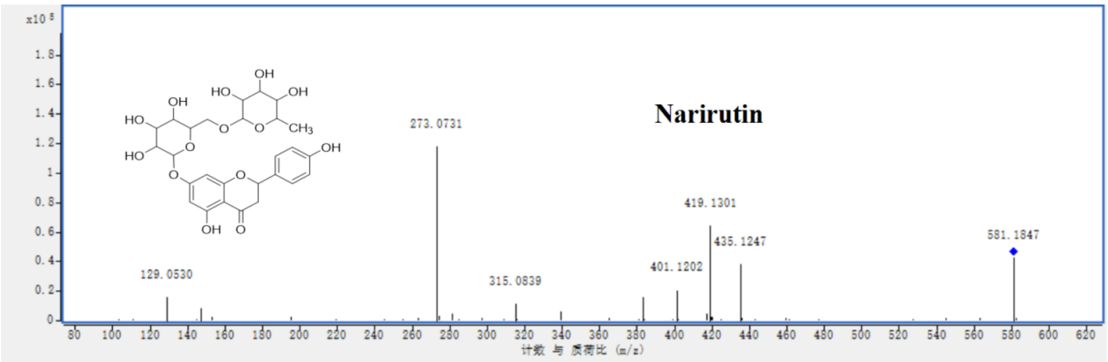

Narirutin
x10 5
273. 0731
419. 1301
435. 1247
401. 1202
315. 0839
129. 0530
581. 1847
HO
OH
CH3
计数 与 质荷比 (m/z)

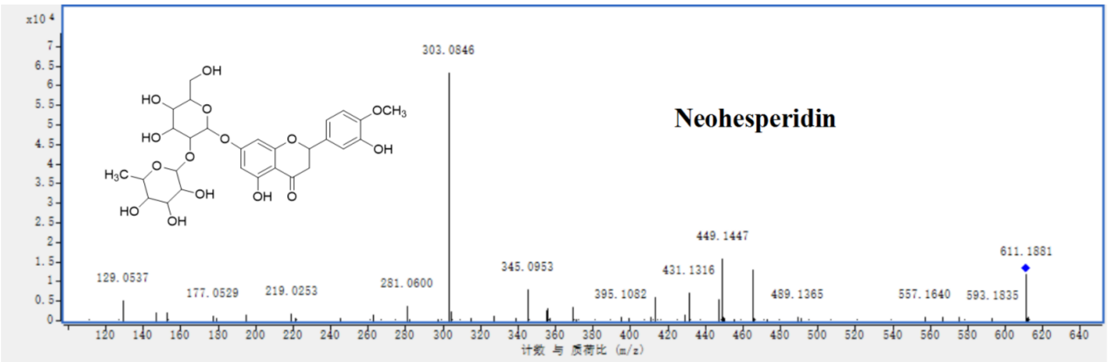

Neohesperidin
x10 4
303. 0846
449. 1447
611. 1881
345. 0953
431. 1316
129. 0537
177. 0529
219. 0253
281. 0600
395. 1082
489. 1365
557. 1640
593. 1835
OCH3
H3C
计数 与 质荷比 (m/z)

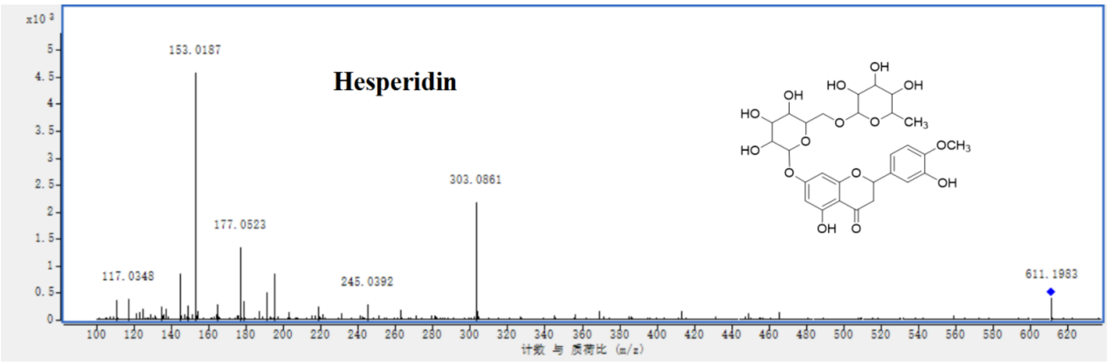

Hesperidin
x10 3
153. 0187
177. 0523
303. 0861
117. 0348
245. 0392
611. 1983
OCH3
CH3
计数 与 质荷比 (m/z)

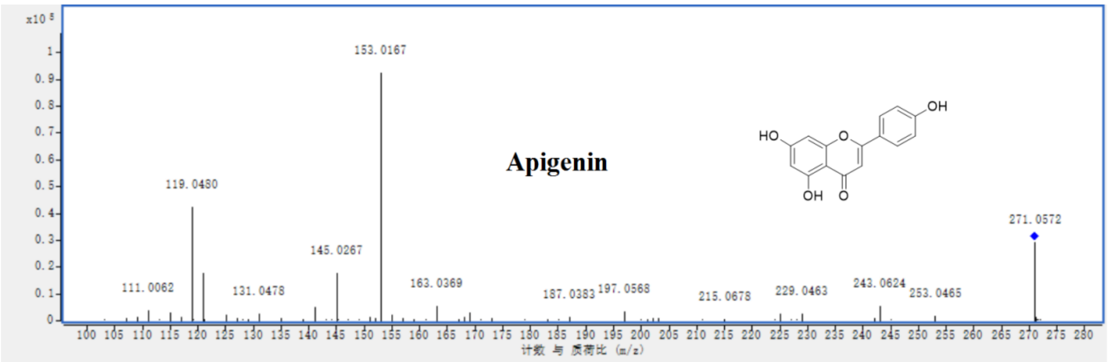

Apigenin
x10 5
153. 0167
119. 0480
145. 0267
111. 0062
131. 0478
163. 0369
187. 0383
197. 0568
215. 0678
229. 0463
243. 0624
253. 0465
271. 0572
HO
OH
计数 与 质荷比 (m/z)

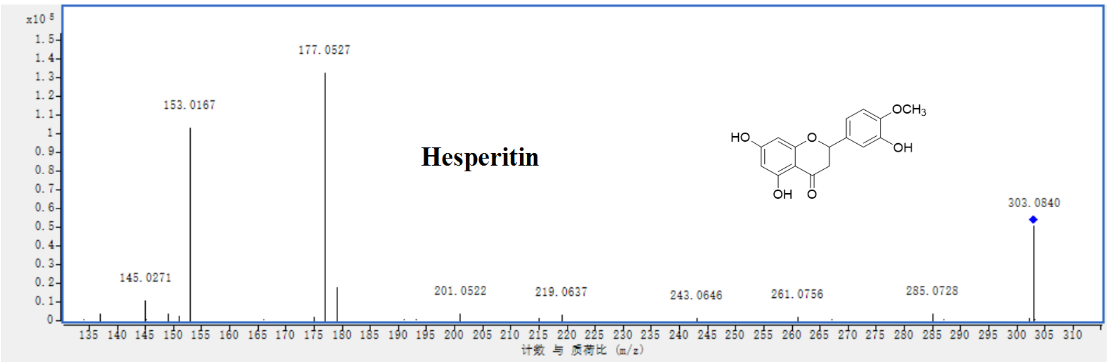
x10 5
Hesperitin
HO
OCH3
OH
OH
O
O
177.0527
153.0167
145.0271
201.0522
219.0637
243.0646
261.0756
285.0728
303.0840
计数 与 质荷比 (m/z)

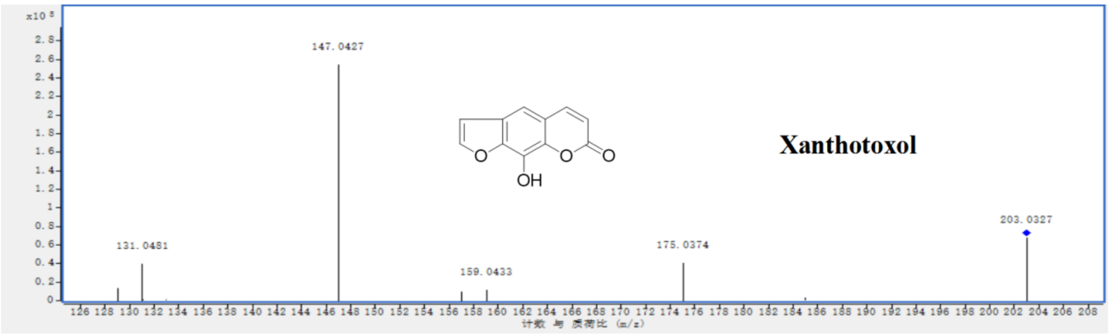
x10 5
Xanthotoxol
O
O
O
OH
147.0427
131.0481
159.0433
175.0374
203.0327
计数 与 质荷比 (m/z)

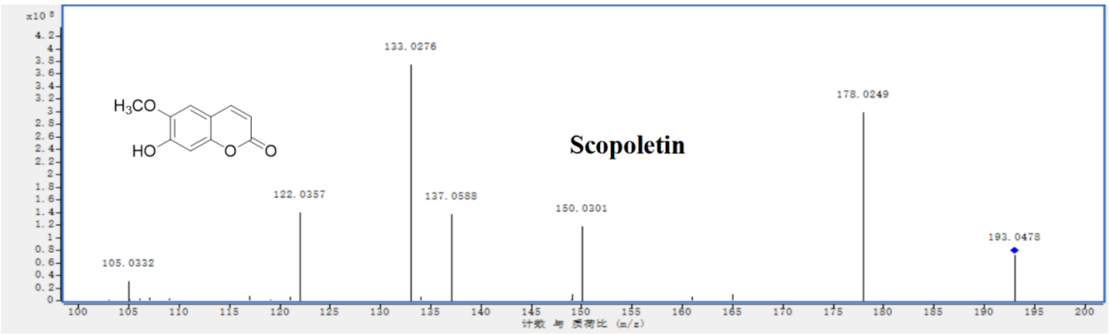
x10 5
Scopoletin
H3CO
HO
O
O
133.0276
178.0249
122.0357
137.0588
150.0301
105.0332
193.0478
计数 与 质荷比 (m/z)

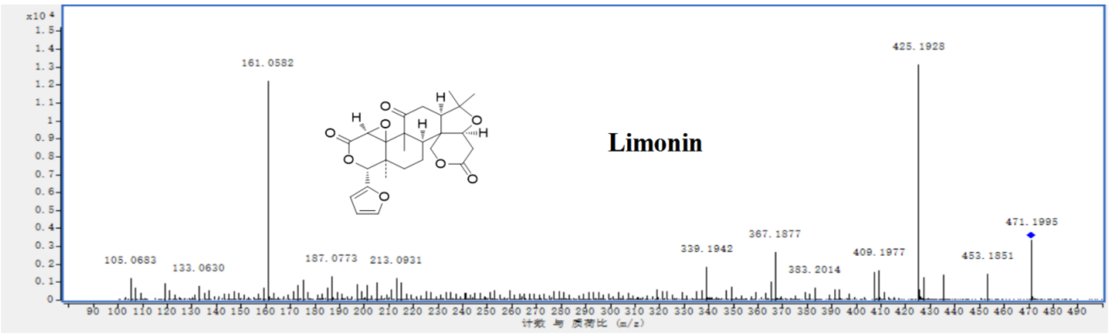
x10 4
Limonin
161.0582
425.1928
471.1995
339.1942
367.1877
383.2014
409.1977
453.1851
105.0683
133.0630
187.0773
213.0931
计数 与 质荷比 (m/z)

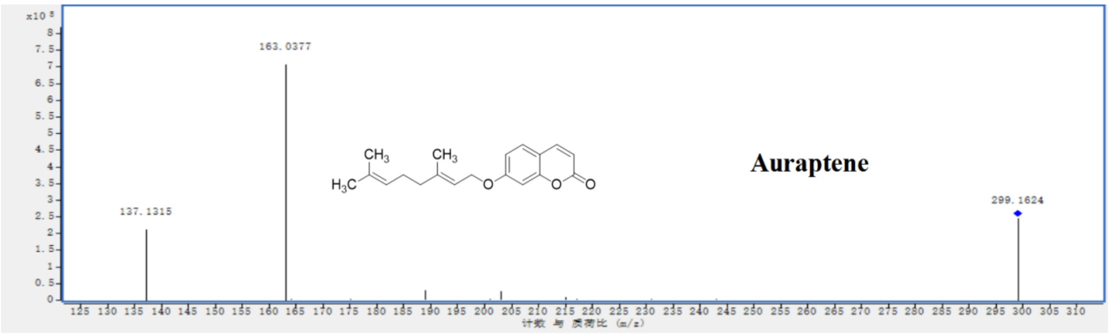
x10 5
Auraptene
CH3
CH3
H3C
O
O
O
163.0377
137.1315
299.1624
计数 与 质荷比 (m/z)

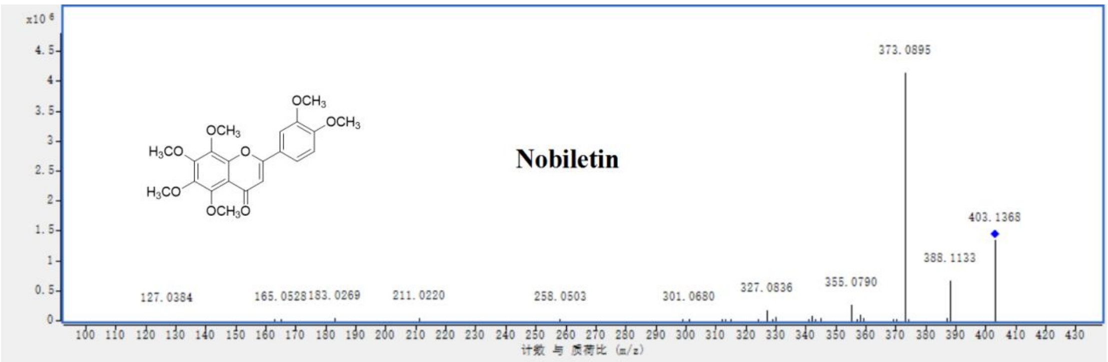

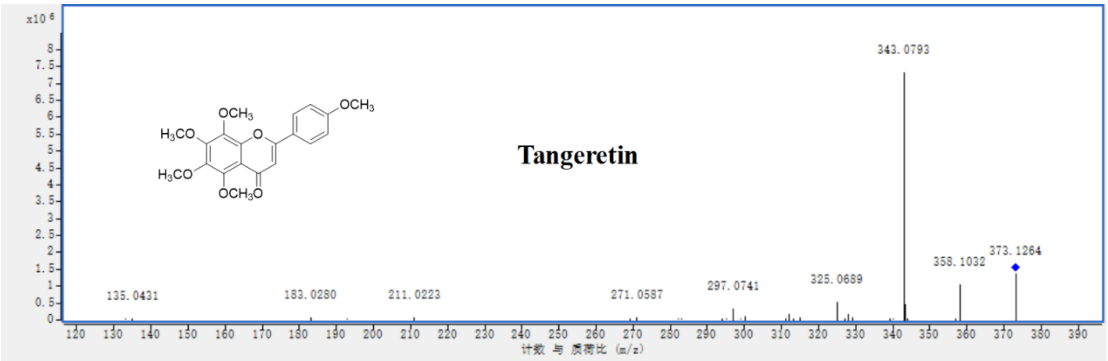

Figure S1. The MS/MS spectra of synephrine (**15**), neoeriocitrin (**36**), poncirin (**39**), eriocitrin (**40**), naringin (**42**), naringenin (**43**), narirutin (**44**), neohesperidin (**51**), hesperidin (**56**), apigenin (**59**), hesperitin (**61**), xanthotoxol (**71**), scopoletin (**76**), limonin (**79**), auraptene (**80**), nobiletin (**82**), and tangeretin (**83**).

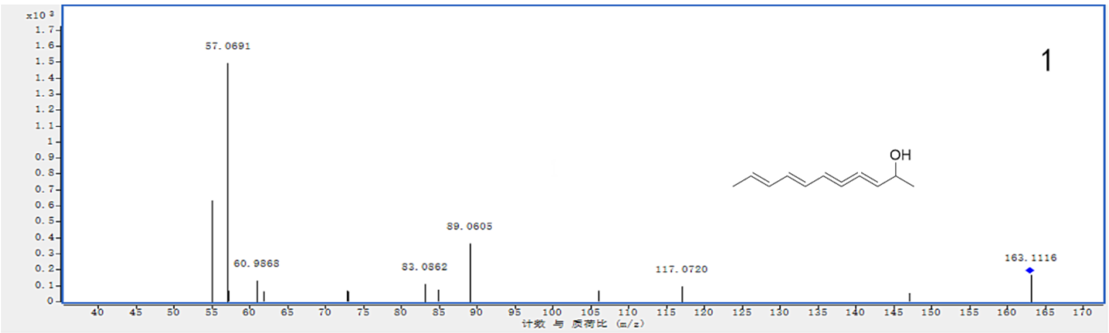

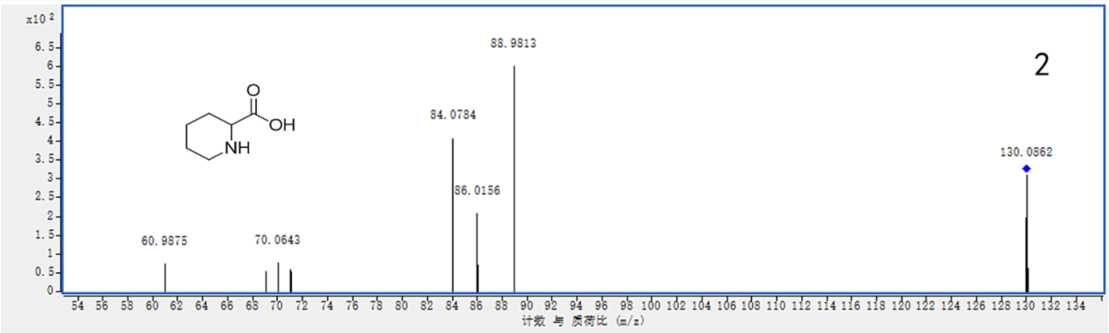

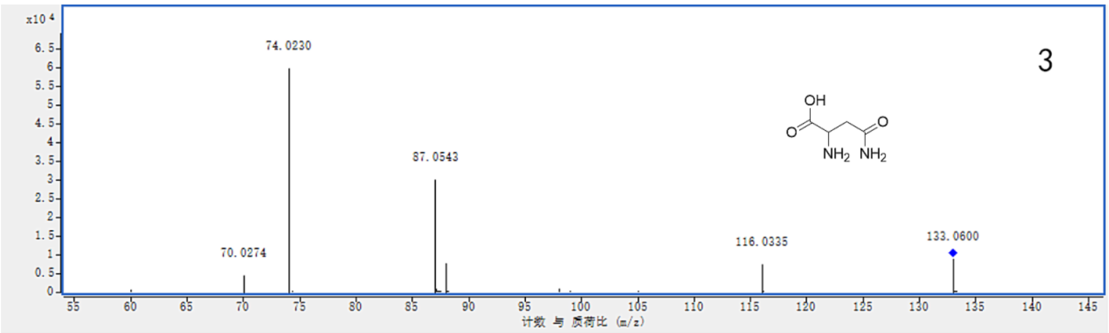
3
x10 4
74.0230
87.0543
70.0274
116.0335
133.0600
OH
O
NH2 NH2
计数 与 质荷比 (m/z)

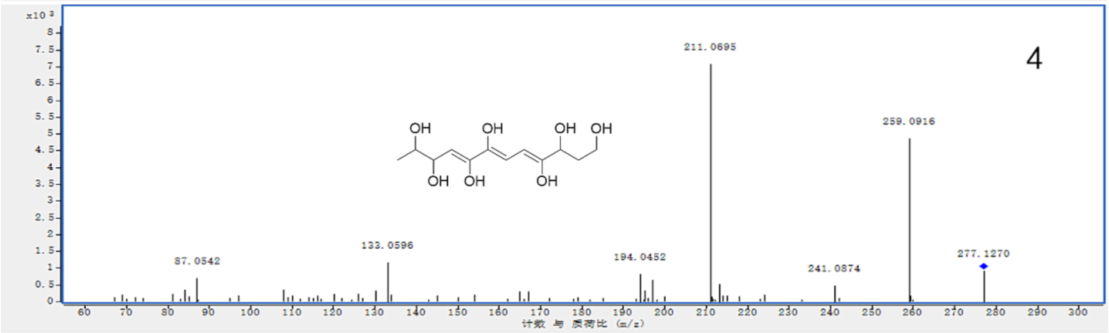
4
x10 3
211.0695
259.0916
277.1270
241.0874
194.0452
133.0596
87.0542
OH OH OH OH
OH OH OH
计数 与 质荷比 (m/z)

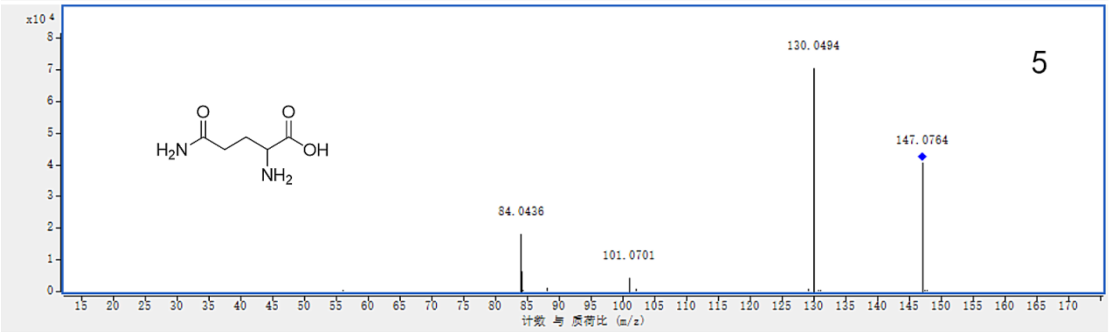
5
x10 4
130.0494
147.0764
84.0436
101.0701
H2N
NH2
OH
计数 与 质荷比 (m/z)

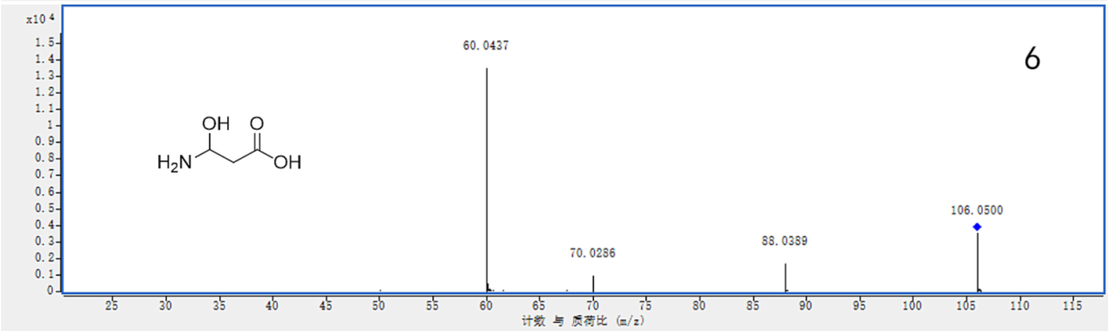
6
x10 4
60.0437
106.0500
88.0389
70.0286
OH
H2N
OH
计数 与 质荷比 (m/z)

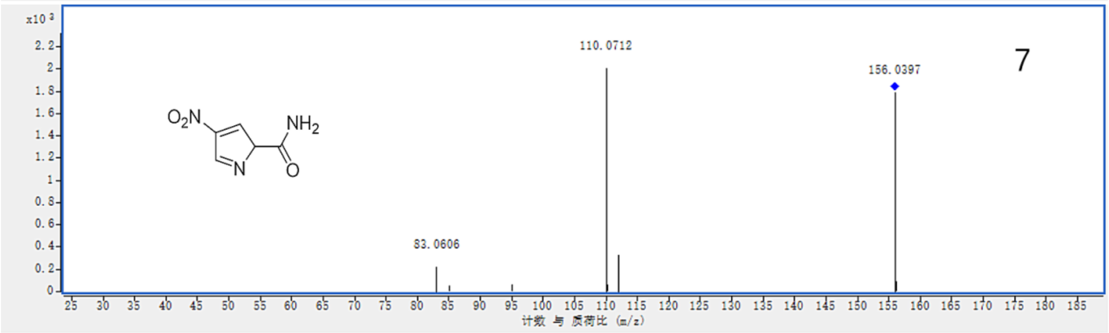
7
x10 3
110.0712
156.0397
83.0606
O2N
NH2
N
计数 与 质荷比 (m/z)

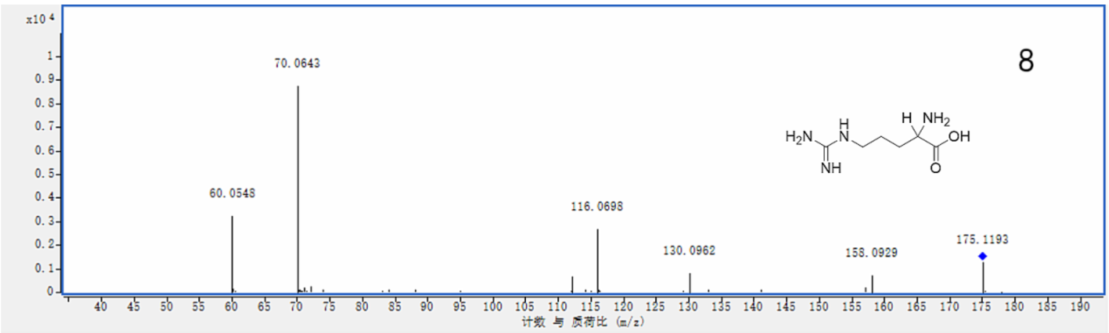
x10 4
8
70.0643
60.0548
116.0698
130.0962
158.0929
175.1193
H_2N
H
N
NH
H
NH_2
OH
O
计数 与 质荷比 (m/z)

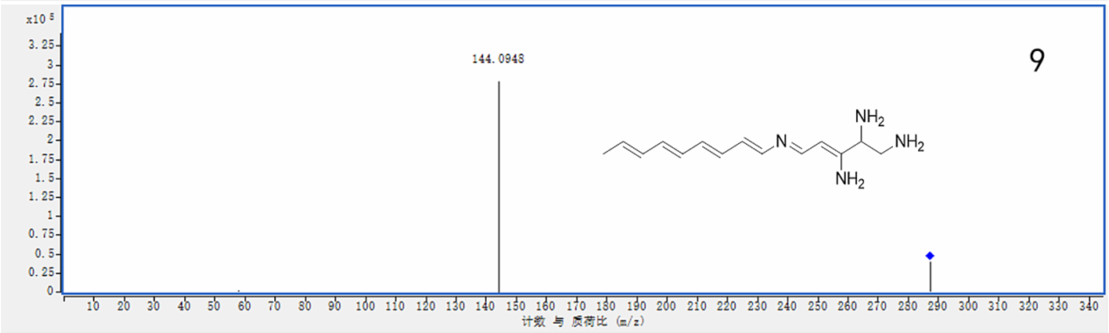
x10 5
9
144.0948
N
NH_2
NH_2
NH_2
计数 与 质荷比 (m/z)

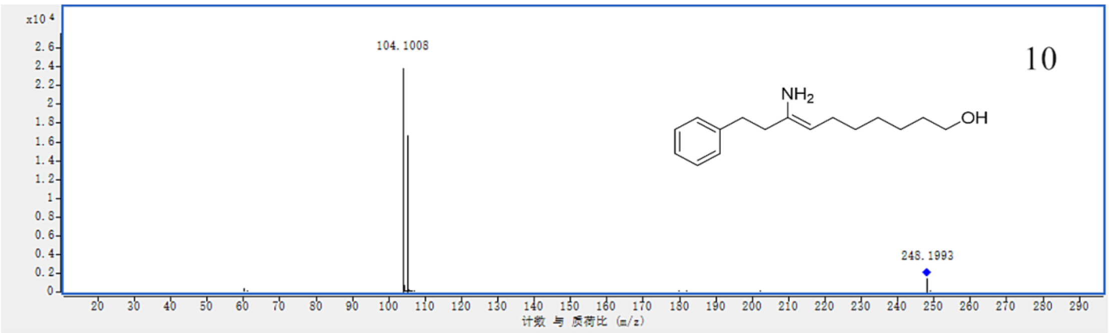
x10 4
10
104.1008
248.1993
NH_2
OH
计数 与 质荷比 (m/z)

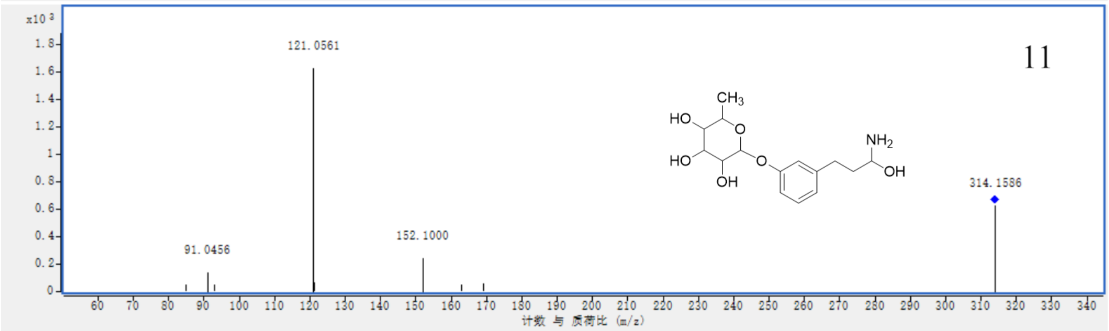
x10 3
11
121.0561
91.0456
152.1000
314.1586
CH_3
HO
O
HO
OH
O
NH_2
OH
计数 与 质荷比 (m/z)

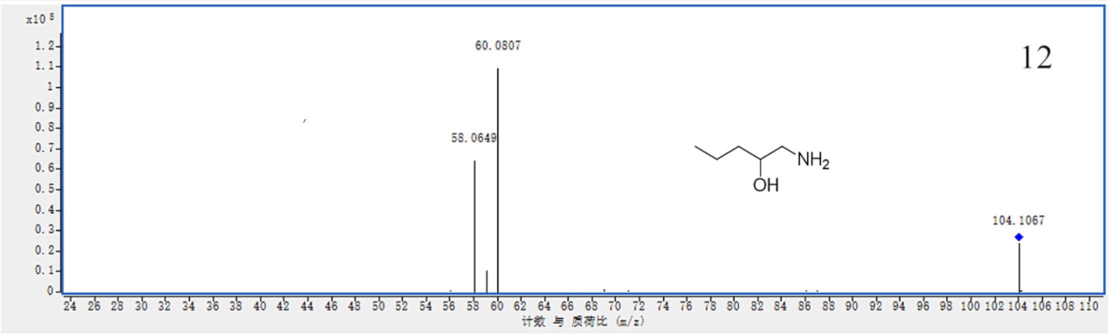
x10 5
12
60.0807
58.0649
104.1067
NH_2
OH
计数 与 质荷比 (m/z)

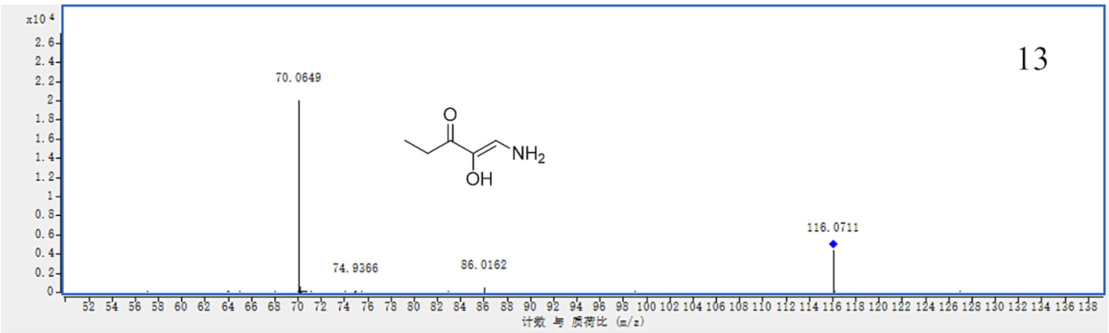
x10 4
13
70.0649
74.9366
86.0162
116.0711
NH2
OH
O
计数 与 质荷比 (m/z)

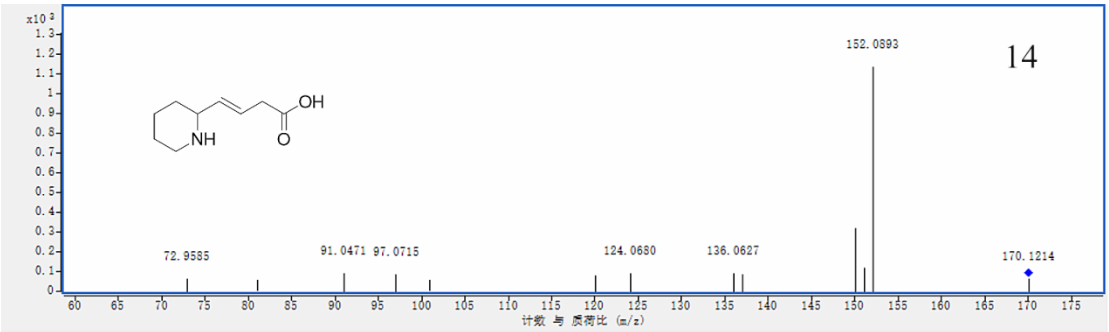
x10 3
14
152.0893
72.9585
91.0471
97.0715
124.0680
136.0627
170.1214
NH
OH
O
计数 与 质荷比 (m/z)

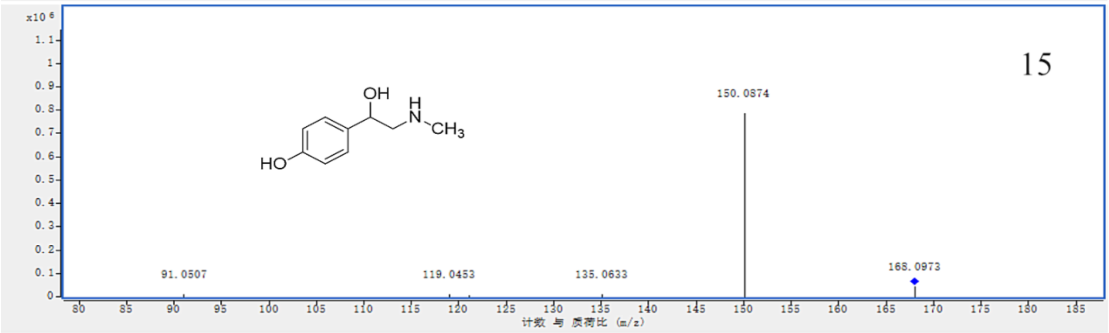
x10 6
15
150.0874
91.0507
119.0453
135.0633
168.0973
OH
H
N
CH3
HO
计数 与 质荷比 (m/z)

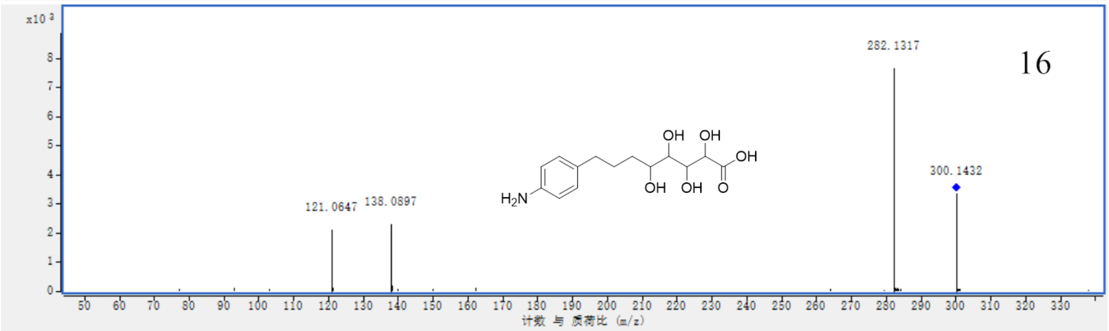
x10 3
16
282.1317
300.1432
121.0647
138.0897
H2N
OH
OH
OH
OH
OH
O
计数 与 质荷比 (m/z)

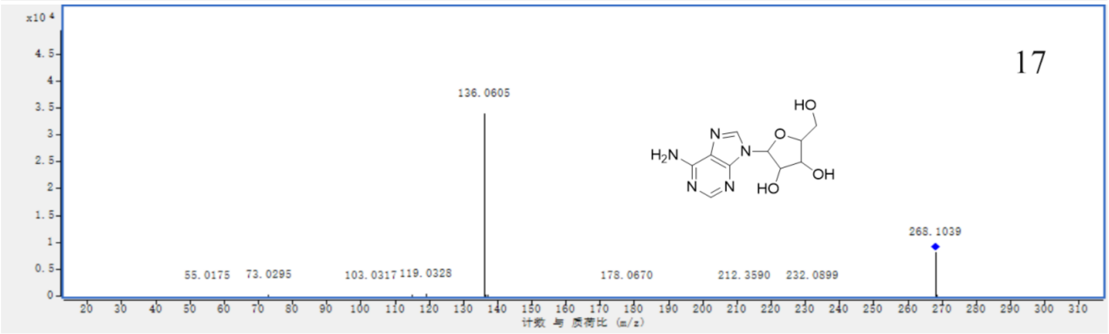
x10 4
17
136.0605
55.0175
73.0295
103.0317
119.0328
178.0670
212.3590
232.0899
268.1039
H2N
HO
OH
HO
计数 与 质荷比 (m/z)

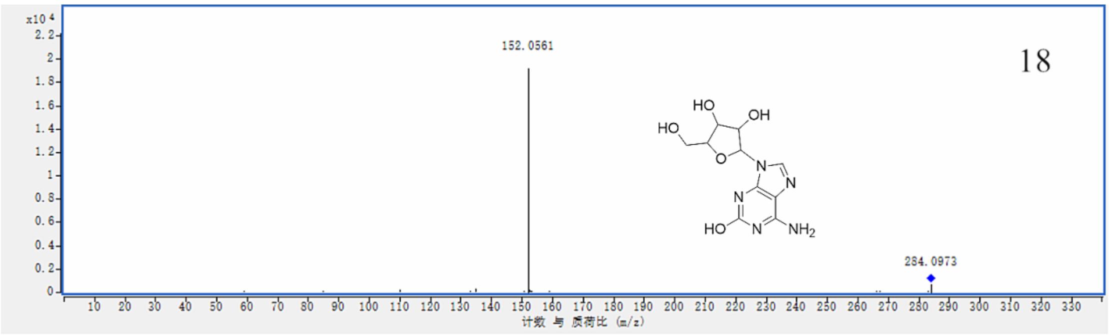
x10 4
152. 0561
18
HO
OH
HO
O
N
N
N
HO
N
NH2
284. 0973
计数 与 质荷比 (m/z)

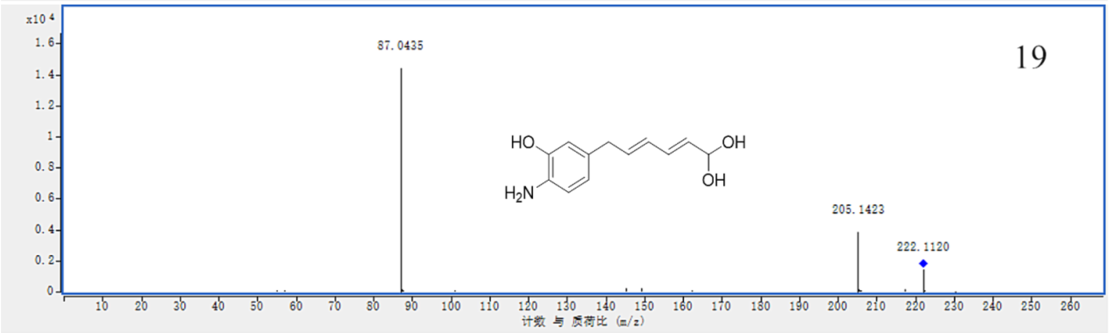
x10 4
87. 0435
19
HO
OH
H2N
OH
205. 1423
222. 1120
计数 与 质荷比 (m/z)

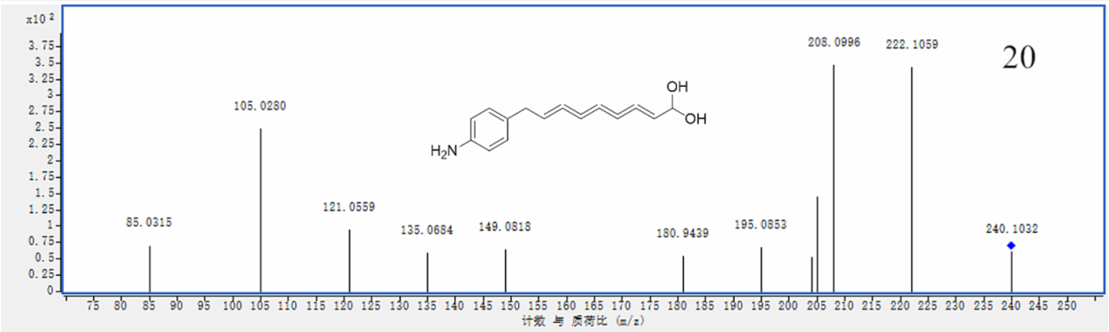
x10 2
208. 0996
222. 1059
20
105. 0280
OH
OH
H2N
121. 0559
85. 0315
135. 0684
149. 0818
180. 9439
195. 0853
240. 1032
计数 与 质荷比 (m/z)

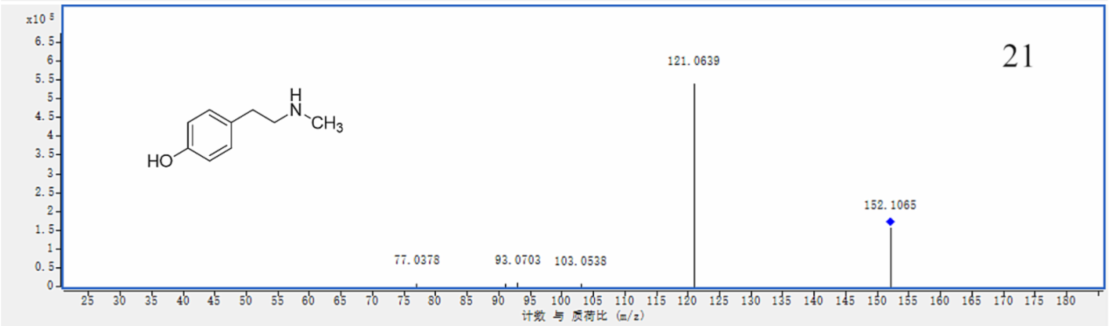
x10 5
121. 0639
21
H
N
CH3
HO
152. 1065
77. 0378
93. 0703
103. 0538
计数 与 质荷比 (m/z)

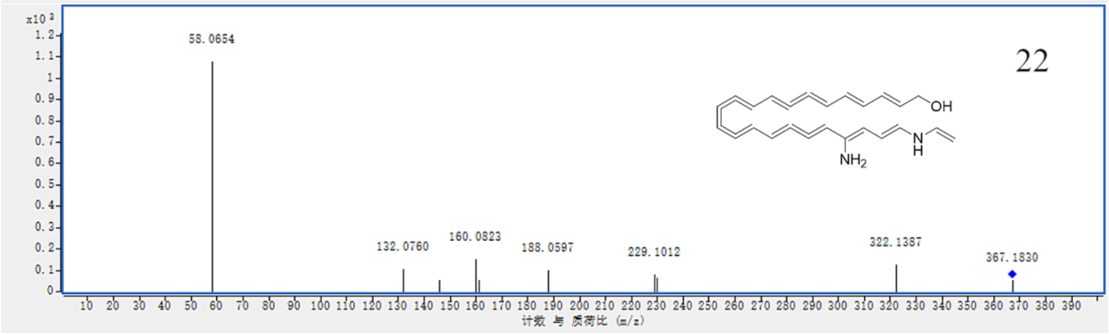
x10 3
58. 0654
22
OH
N
H
NH2
132. 0760
160. 0823
188. 0597
229. 1012
322. 1387
367. 1830
计数 与 质荷比 (m/z)

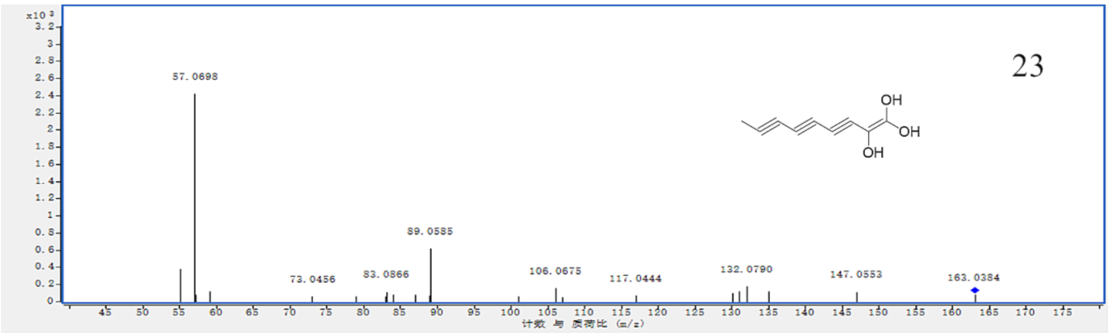
x10 3
23
57.0698
89.0585
73.0456
83.0866
106.0675
117.0444
132.0790
147.0553
163.0384
OH
OH
OH
计数 与 质荷比 (m/z)

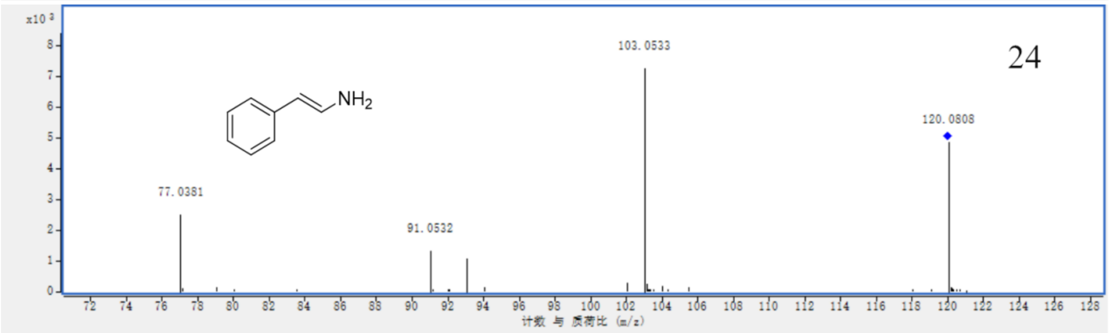
x10 3
24
NH_2
103.0533
120.0808
77.0381
91.0532
计数 与 质荷比 (m/z)

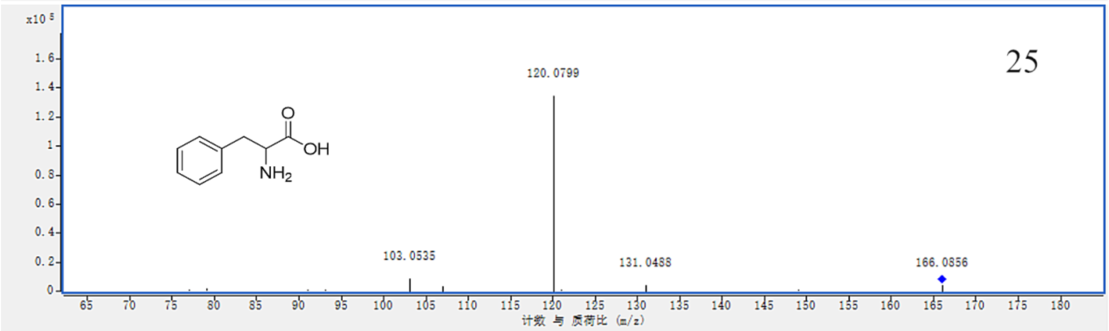
x10 5
25
O
OH
NH_2
120.0799
103.0535
131.0488
166.0856
计数 与 质荷比 (m/z)

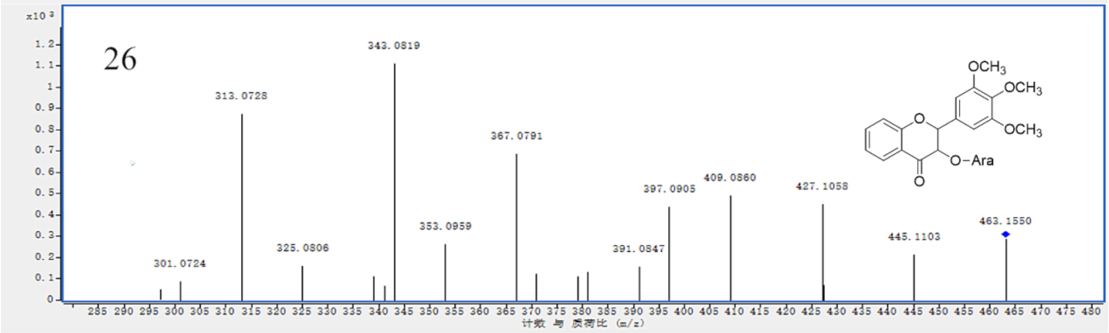
x10 3
26
OCH_3
OCH_3
OCH_3
O
O–Ara
O
343.0819
313.0728
367.0791
397.0905
409.0860
427.1058
353.0959
391.0847
445.1103
463.1550
325.0806
301.0724
计数 与 质荷比 (m/z)

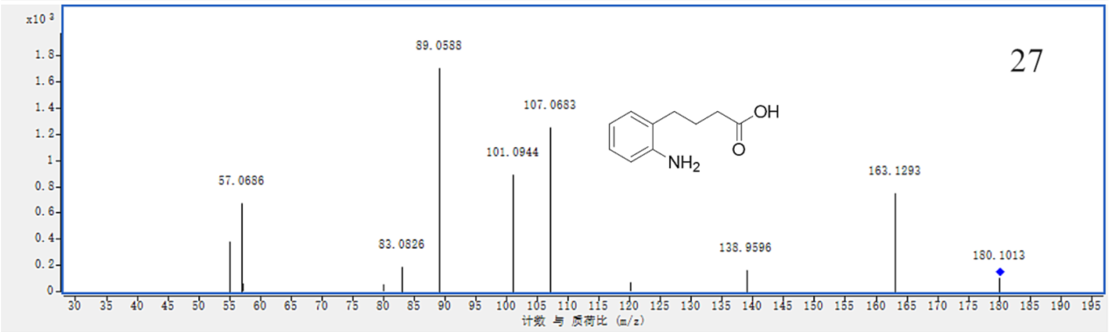
x10 3
27
OH
O
NH_2
89.0588
107.0683
101.0944
57.0686
163.1293
83.0826
138.9596
180.1013
计数 与 质荷比 (m/z)

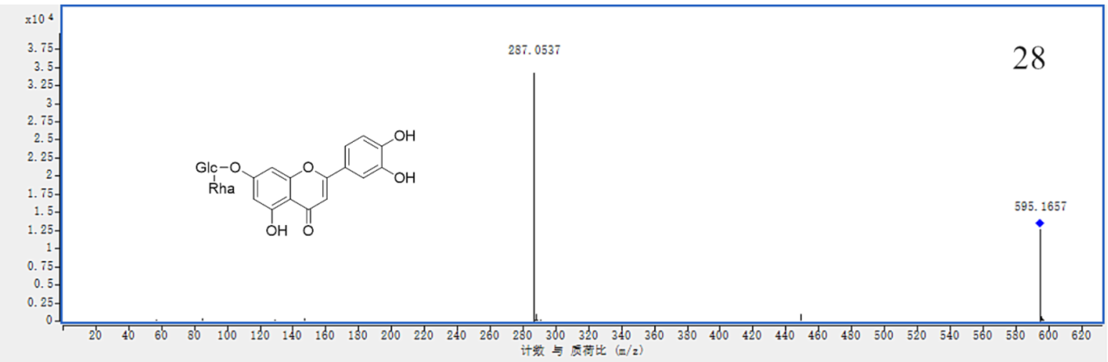
x10 4
287.0537
595.1657
28
Glc-O
Rha
OH
OH
OH
O
O
计数 与 质荷比 (m/z)

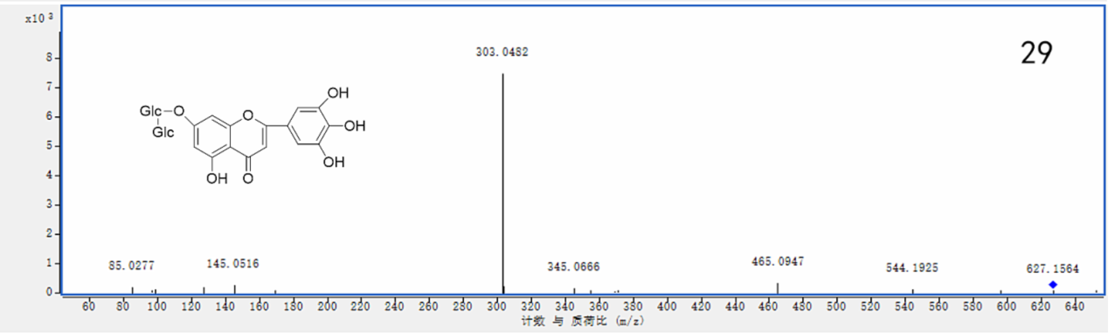
x10 3
303.0482
85.0277
145.0516
345.0666
465.0947
544.1925
627.1564
29
Glc-O
Glc
OH
OH
OH
OH
O
O
计数 与 质荷比 (m/z)

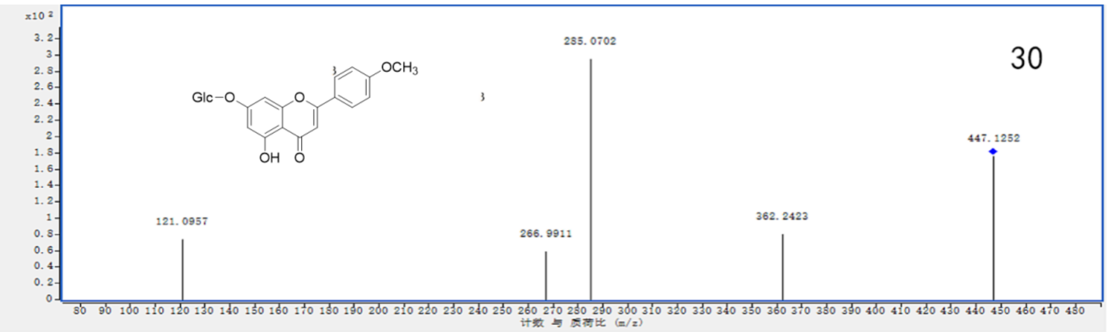
x10 2
285.0702
447.1252
121.0957
266.9911
362.2423
30
Glc-O
OCH_3
OH
O
O
计数 与 质荷比 (m/z)

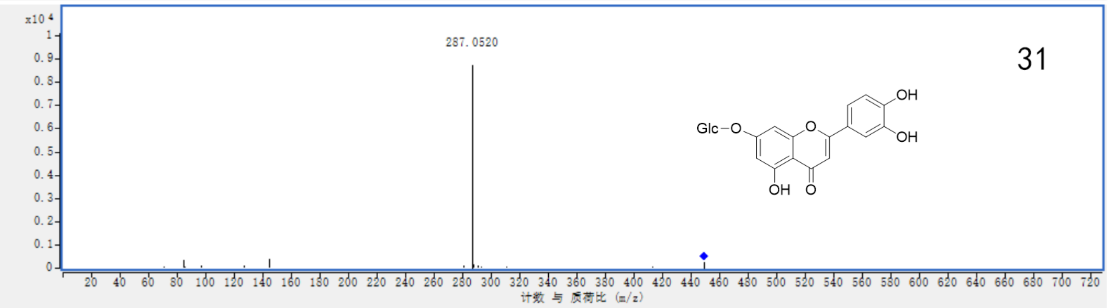
x10 4
287.0520
31
Glc-O
OH
OH
OH
O
O
计数 与 质荷比 (m/z)

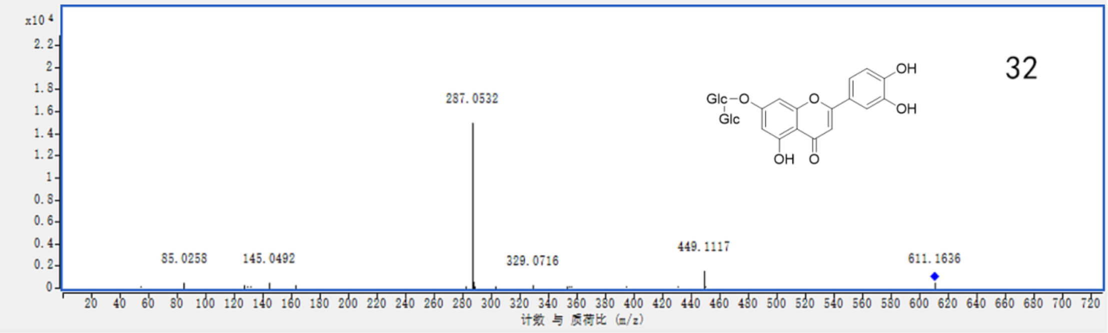
x10 4
287.0532
85.0258
145.0492
329.0716
449.1117
611.1636
32
Glc-O
Glc
OH
OH
OH
O
O
计数 与 质荷比 (m/z)

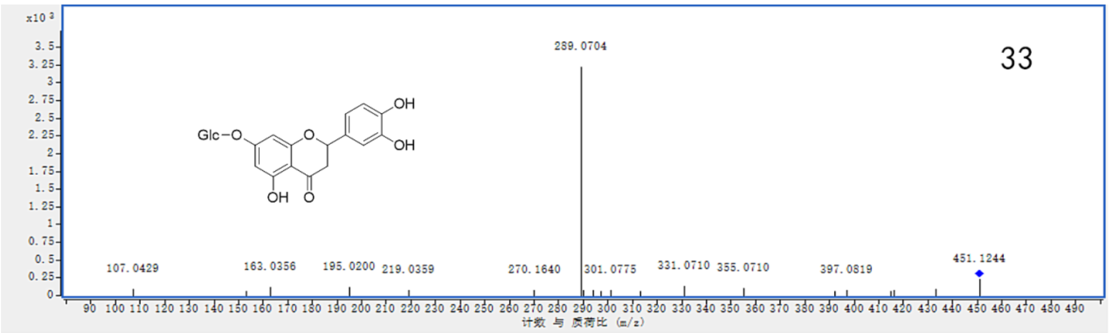
x10 3
33
289.0704
Glc–O
OH
OH
OH
O
107.0429
163.0356
195.0200
219.0359
270.1640
301.0775
331.0710
355.0710
397.0819
451.1244
计数 与 质荷比 (m/z)

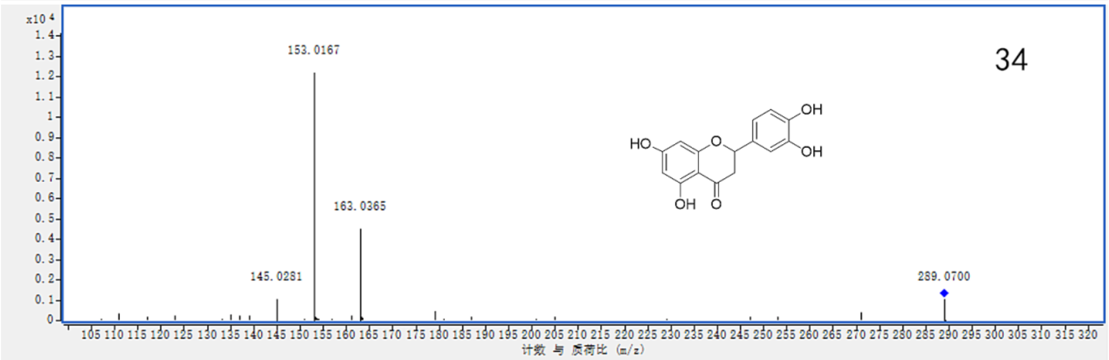
x10 4
34
153.0167
HO
OH
OH
OH
O
163.0365
145.0281
289.0700
计数 与 质荷比 (m/z)

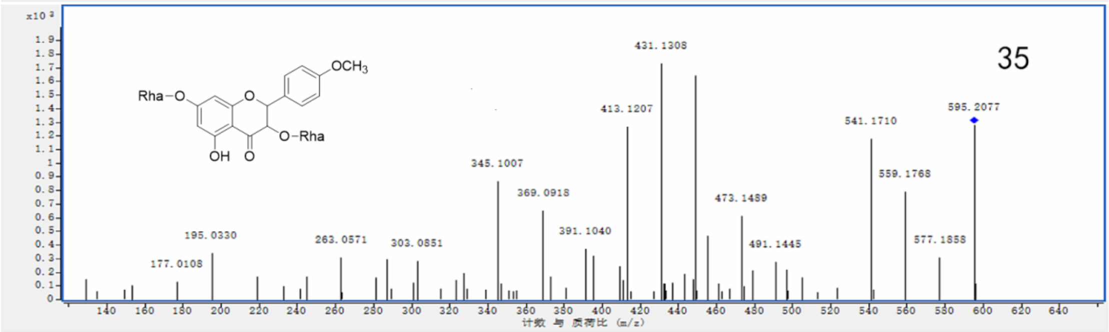
x10 3
35
431.1308
Rha–O
OCH3
O–Rha
OH
O
413.1207
541.1710
595.2077
345.1007
369.0918
559.1768
473.1489
391.1040
195.0330
263.0571
303.0851
491.1445
577.1858
177.0108
计数 与 质荷比 (m/z)

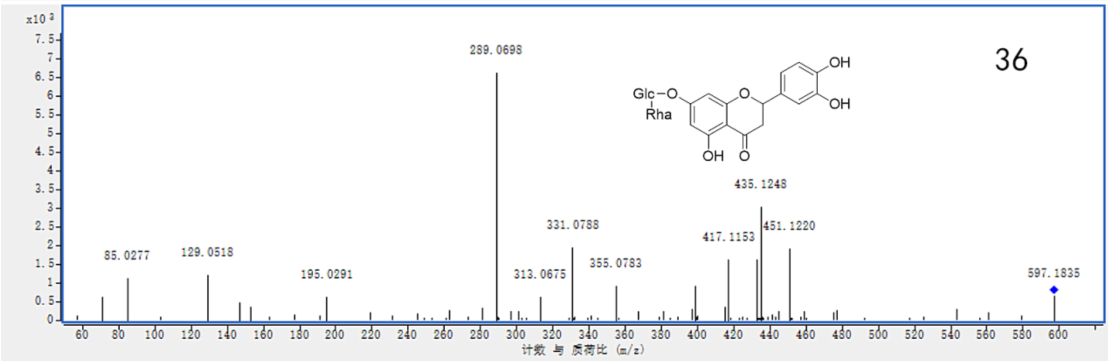
x10 3
36
289.0698
Glc–O
Rha
OH
OH
OH
O
435.1248
331.0788
417.1153
451.1220
85.0277
129.0518
355.0783
195.0291
313.0675
597.1835
计数 与 质荷比 (m/z)

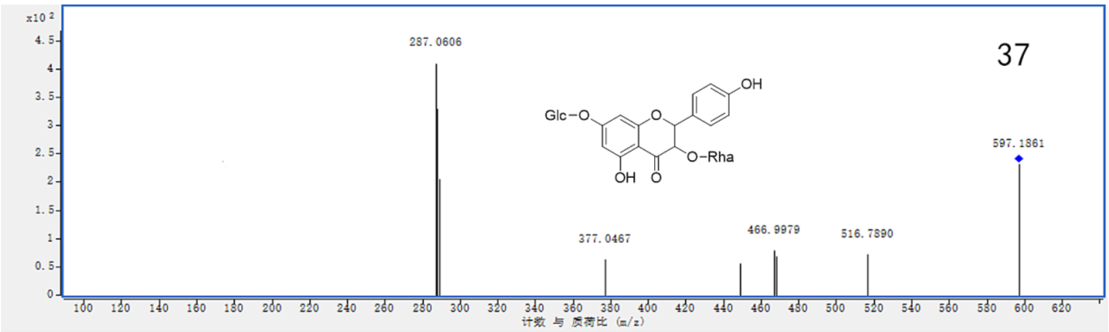
x10 2
37
287.0606
OH
Glc–O
O–Rha
OH
O
597.1861
377.0467
466.9979
516.7890
计数 与 质荷比 (m/z)

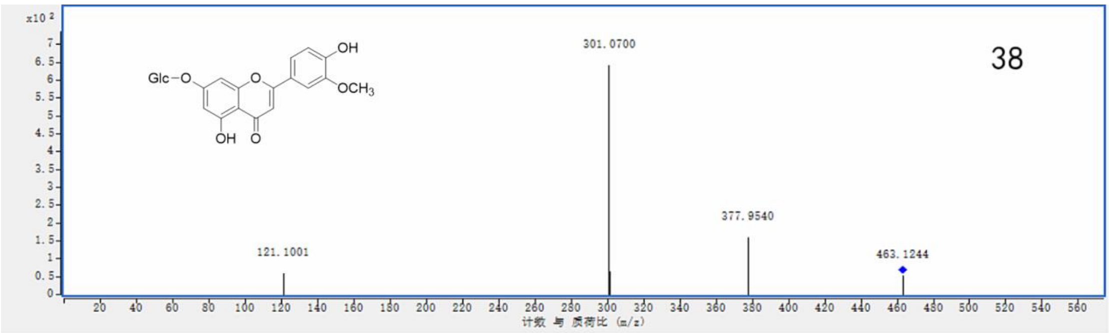
x10 2
38
Glc−O
OH
OCH3
OH O
301.0700
377.9540
121.1001
463.1244
计数 与 质荷比 (m/z)

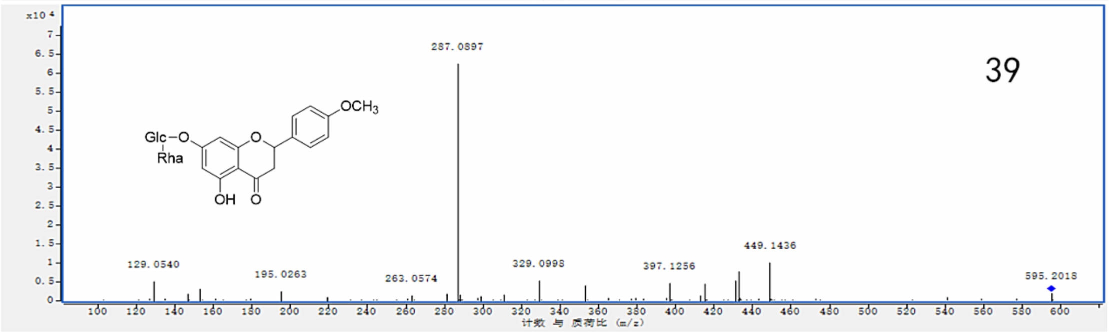
x10 4
39
OCH3
Glc−O
Rha
OH O
287.0897
449.1436
129.0540
195.0263
263.0574
329.0998
397.1256
595.2018
计数 与 质荷比 (m/z)

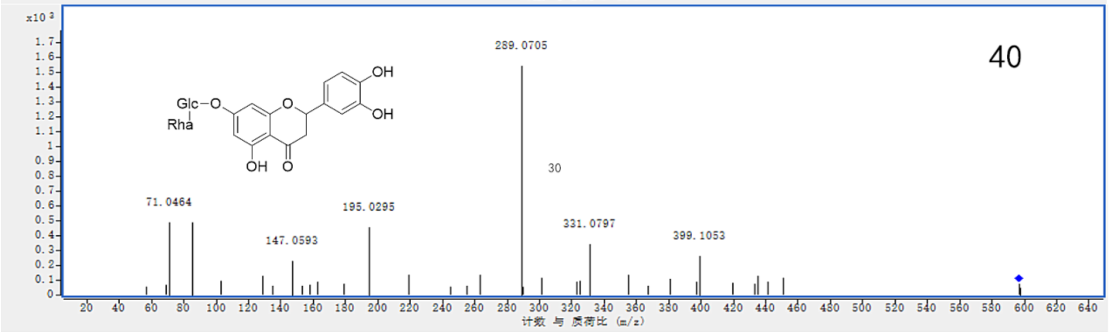
x10 3
40
OH
Glc−O
Rha
OH
OH O
289.0705
30
71.0464
195.0295
147.0593
331.0797
399.1053
计数 与 质荷比 (m/z)

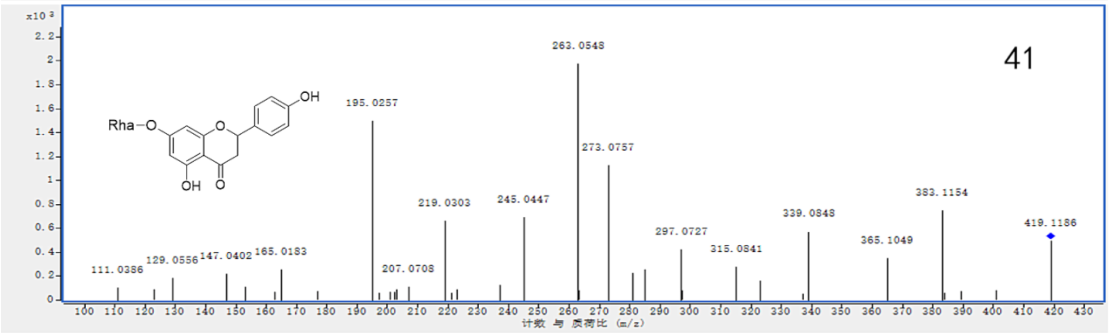
x10 3
41
OH
Rha−O
OH O
263.0548
195.0257
273.0757
219.0303
245.0447
339.0848
383.1154
419.1186
297.0727
315.0541
365.1049
111.0386
129.0556
147.0402
165.0183
207.0708
计数 与 质荷比 (m/z)

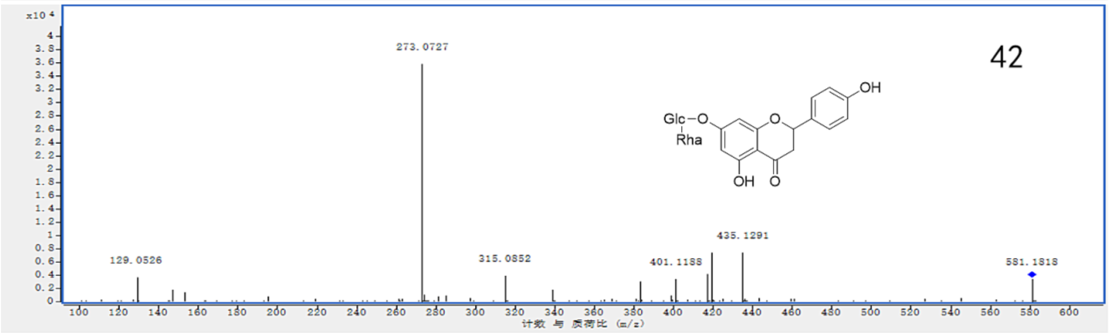
x10 4
42
273.0727
OH
Glc−O
Rha
OH O
129.0526
315.0852
401.1188
435.1291
581.1818
计数 与 质荷比 (m/z)

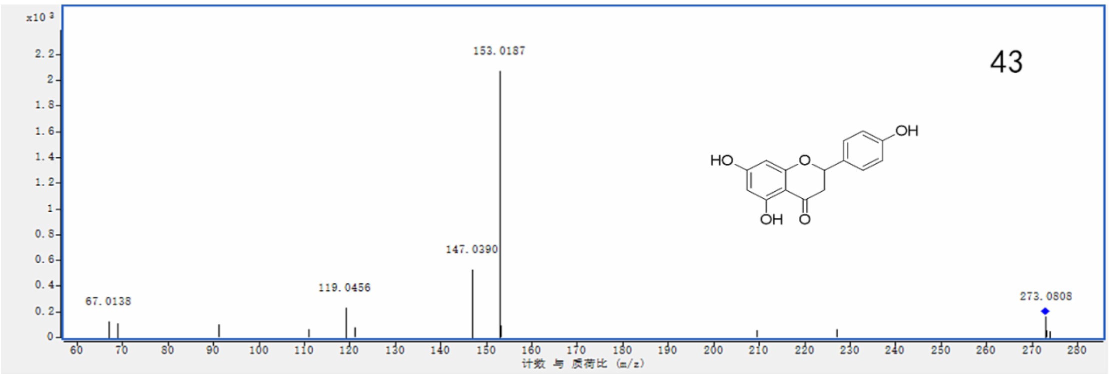
43
153.0187
147.0390
119.0456
67.0138
273.0808
HO
OH
OH
O
O
x10 3
计数 与 质荷比 (m/z)

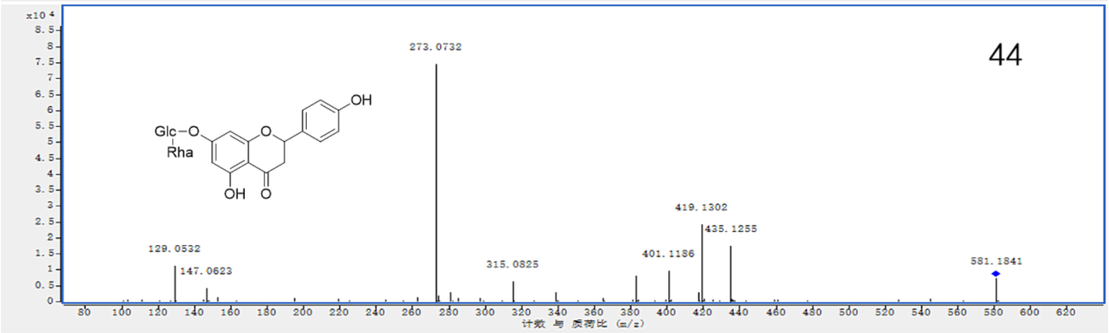
44
273.0732
419.1302
435.1255
401.1186
315.0825
129.0532
147.0623
581.1841
Glc–O
Rha
OH
OH
O
O
x10 4
计数 与 质荷比 (m/z)

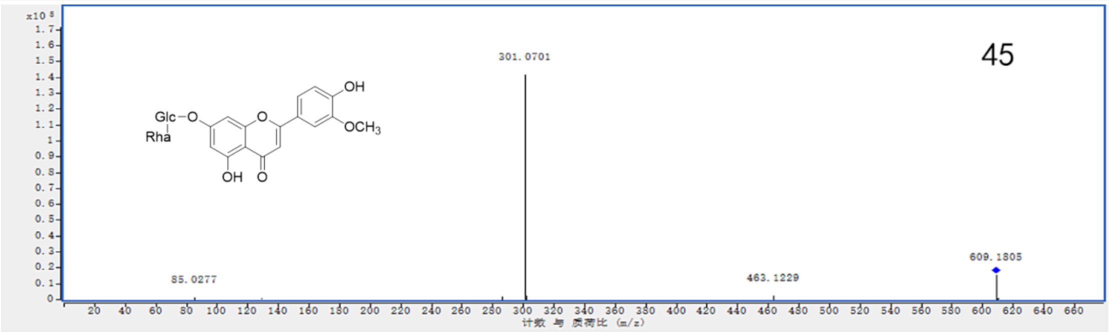
45
301.0701
609.1805
463.1229
85.0277
Glc–O
Rha
OH
OCH3
OH
O
O
x10 5
计数 与 质荷比 (m/z)

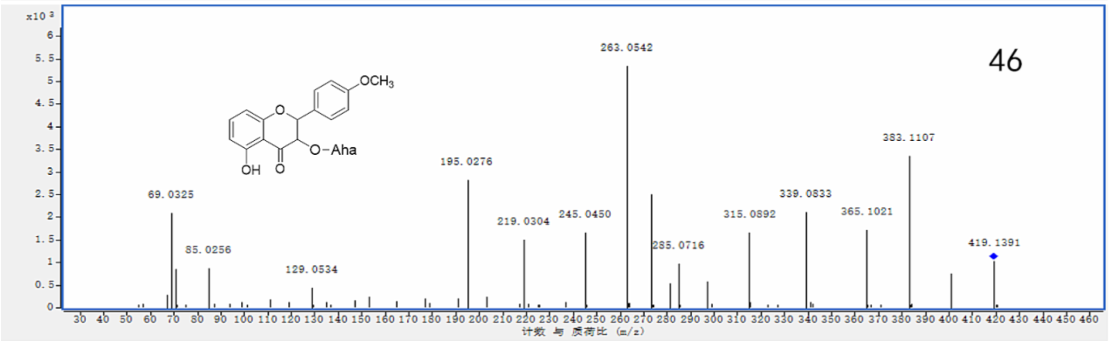
46
263.0542
383.1107
195.0276
69.0325
339.0833
365.1021
219.0304
245.0450
315.0892
85.0256
285.0716
419.1391
129.0534
OCH3
O–Aha
OH
O
O
x10 3
计数 与 质荷比 (m/z)

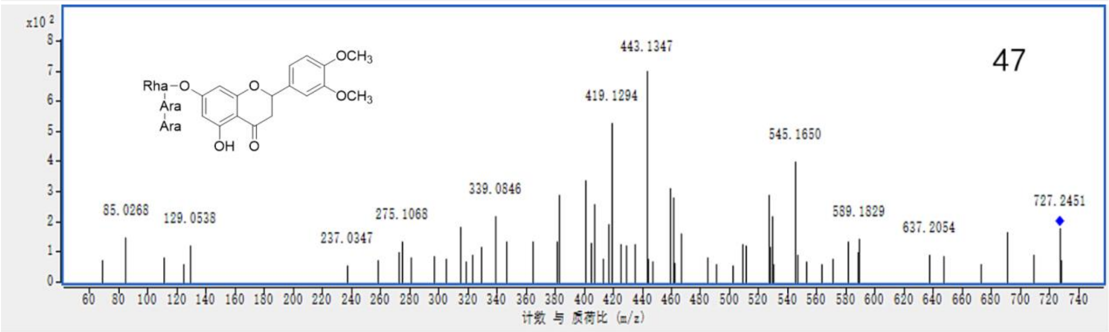
47
443.1347
419.1294
545.1650
339.0846
85.0268
129.0538
275.1068
237.0347
589.1829
637.2054
727.2451
Rha–O
Ara
Ara
OCH3
OCH3
OH
O
O
x10 2
计数 与 质荷比 (m/z)

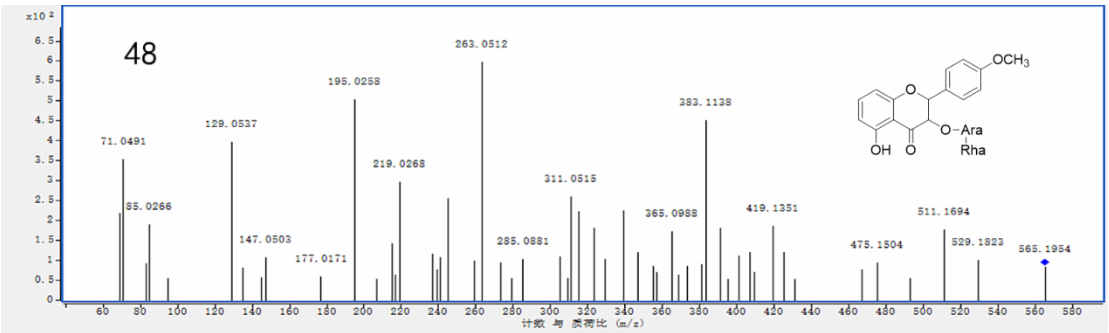
48
x10 2
71.0491
85.0266
129.0537
147.0503
177.0171
195.0258
219.0268
263.0512
285.0881
311.0515
365.0988
383.1138
419.1351
475.1504
511.1694
529.1823
565.1954
OCH3
O
OH O
O–Ara
Rha
计数 与 质荷比 (m/z)

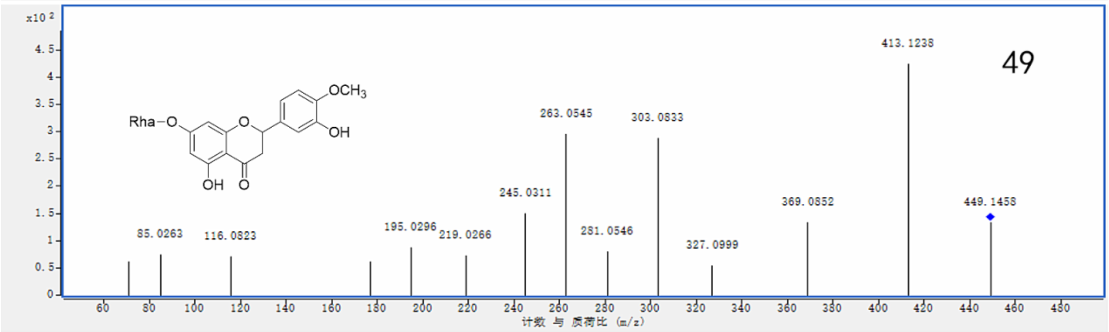
49
x10 2
85.0263
116.0823
195.0296
219.0266
245.0311
263.0545
281.0546
303.0833
327.0999
369.0852
413.1238
449.1458
Rha–O
OCH3
OH
OH O
计数 与 质荷比 (m/z)

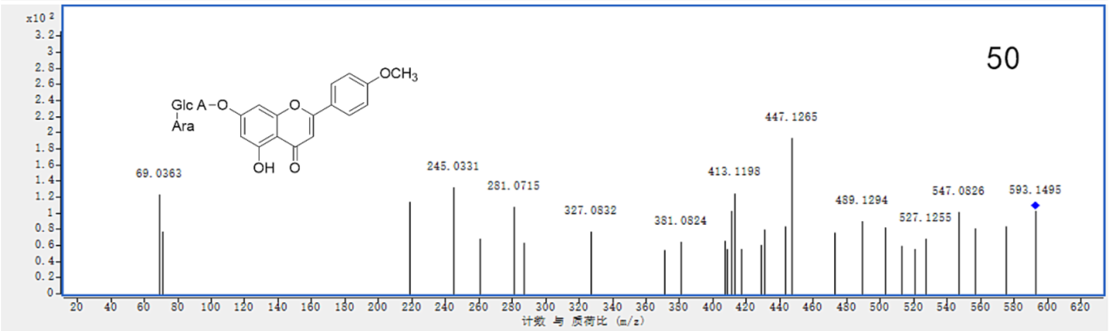
50
x10 2
69.0363
245.0331
281.0715
327.0832
381.0824
413.1198
447.1265
489.1294
527.1255
547.0826
593.1495
Glc A–O
Ara
OCH3
OH O
计数 与 质荷比 (m/z)

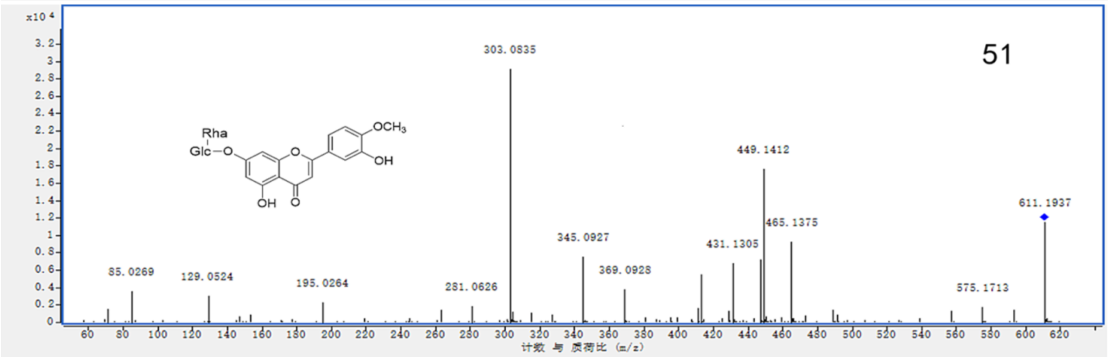
51
x10 4
85.0269
129.0524
195.0264
281.0626
303.0835
345.0927
369.0928
431.1305
449.1412
465.1375
575.1713
611.1937
Rha
Glc–O
OCH3
OH
OH O
计数 与 质荷比 (m/z)

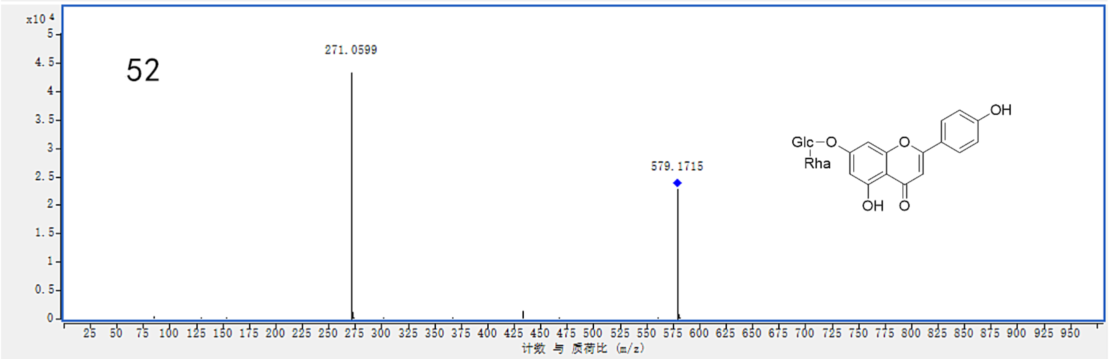
52
x10 4
271.0599
579.1715
Glc–O
Rha
OH
OH O
计数 与 质荷比 (m/z)

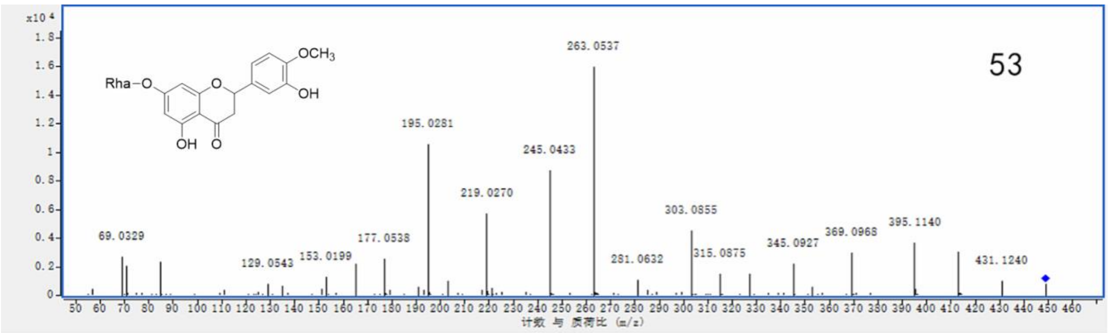
53
x10 4
69.0329
129.0543
153.0199
177.0538
195.0281
219.0270
245.0433
263.0537
281.0632
303.0855
315.0875
345.0927
369.0968
395.1140
431.1240
Rha–O
OCH3
OH
OH O
计数 与 质荷比 (m/z)

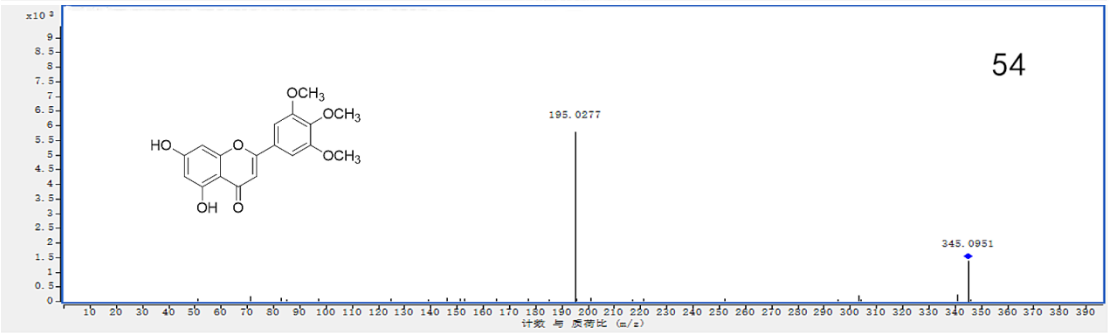
54
x10 3
195.0277
345.0951
HO
OH O
OCH3
OCH3
OCH3
计数 与 质荷比 (m/z)

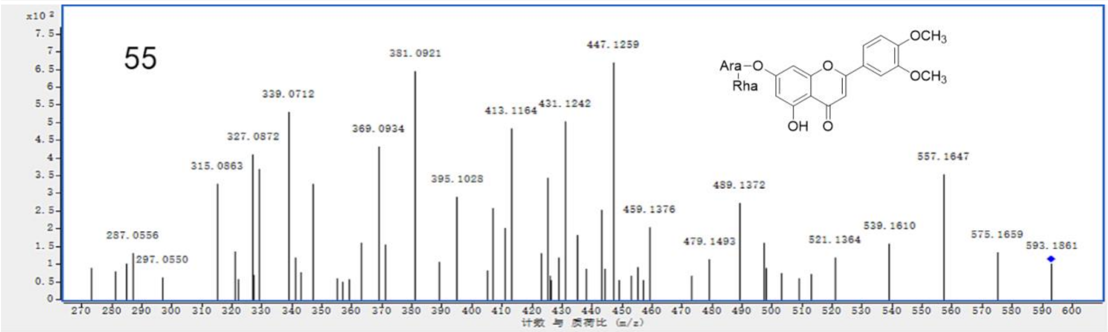
55
x10 2
287.0556
297.0550
315.0863
327.0872
339.0712
369.0934
381.0921
395.1028
413.1164
431.1242
447.1259
459.1376
479.1493
489.1372
521.1364
539.1610
557.1647
575.1659
593.1861
Ara–O
Rha
OCH3
OCH3
OH O
计数 与 质荷比 (m/z)

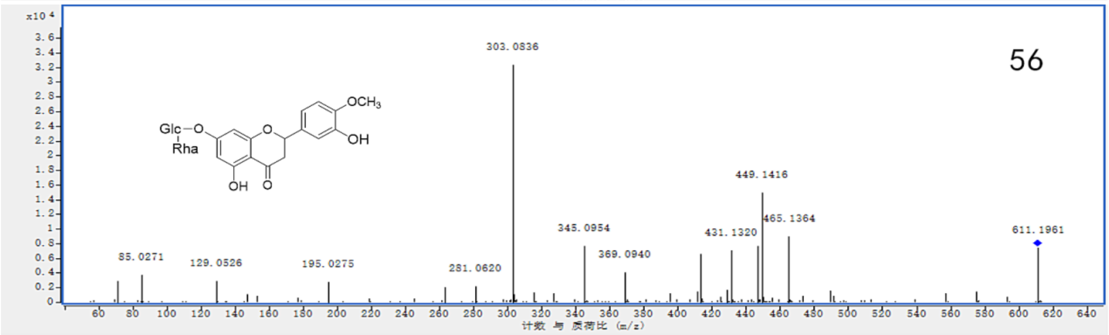
56
x10 4
85.0271
129.0526
195.0275
281.0620
303.0836
345.0954
369.0940
431.1320
449.1416
465.1364
611.1961
Glc–O
Rha
OCH3
OH
OH O
计数 与 质荷比 (m/z)

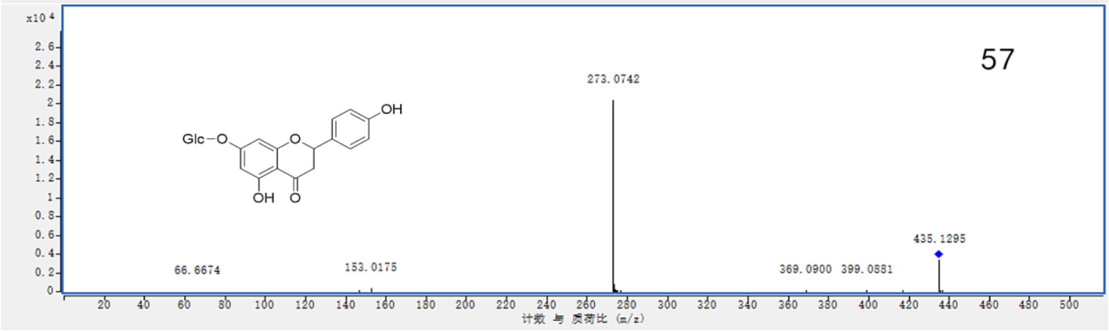
57
x10 4
66.6674
153.0175
273.0742
369.0900
399.0881
435.1295
Glc–O
OH
OH O
计数 与 质荷比 (m/z)

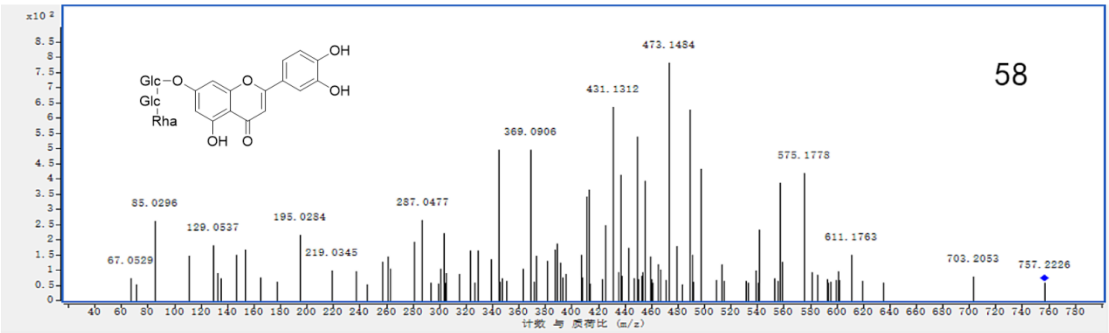
58
x10 2
Glc–O
Glc
Rha
OH
OH
OH O
67.0529
85.0296
129.0537
195.0284
219.0345
287.0477
369.0906
431.1312
473.1484
575.1778
611.1763
703.2053
757.2226
计数 与 质荷比 (m/z)

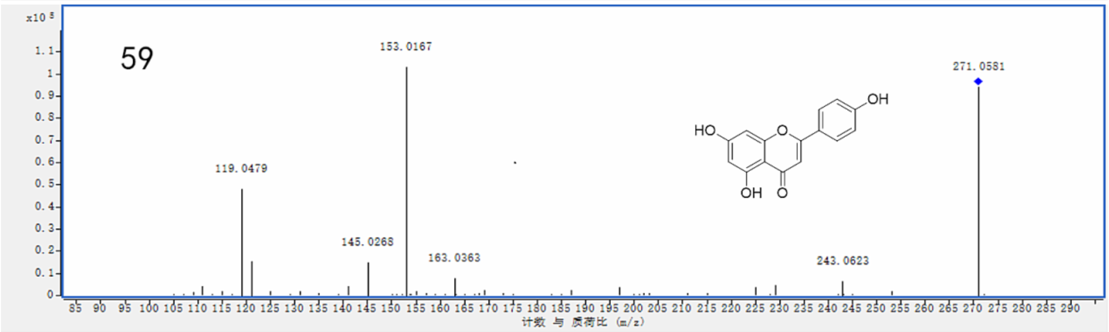
59
x10 5
HO
OH
OH O
119.0479
145.0268
153.0167
163.0363
243.0623
271.0581
计数 与 质荷比 (m/z)

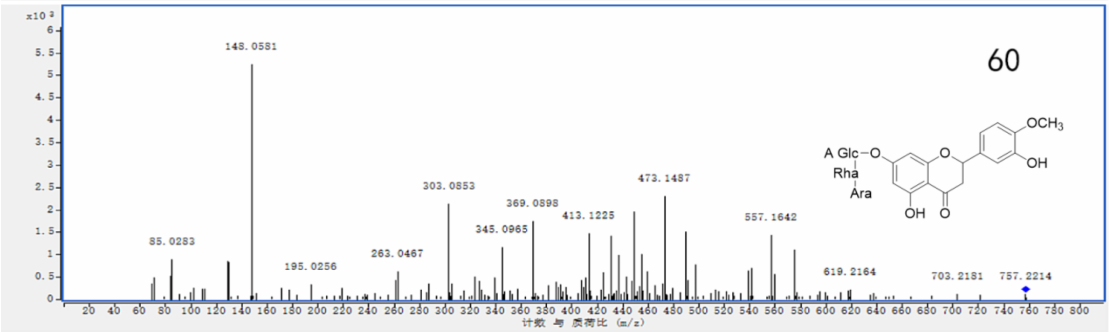
60
x10 3
A Glc–O
Rha
Ara
OCH3
OH
OH O
85.0283
148.0581
195.0256
263.0467
303.0853
345.0965
369.0898
413.1225
473.1487
557.1642
619.2164
703.2181
757.2214
计数 与 质荷比 (m/z)

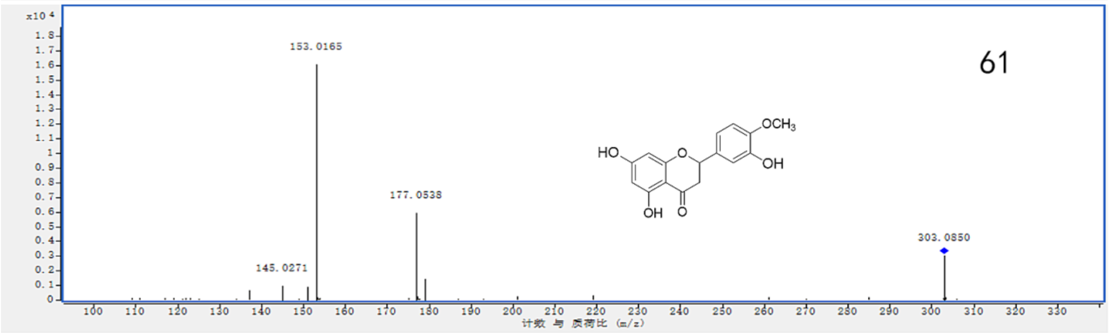
61
x10 4
HO
OCH3
OH
OH O
145.0271
153.0165
177.0538
303.0850
计数 与 质荷比 (m/z)

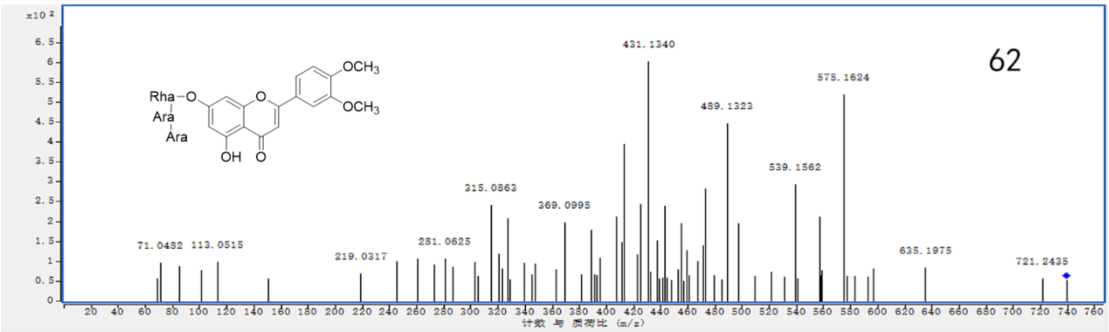
62
x10 2
Rha–O
Ara
Ara
OCH3
OCH3
OH O
71.0482
113.0515
219.0317
281.0625
315.0863
369.0995
431.1340
489.1323
539.1562
575.1624
635.1975
721.2435
计数 与 质荷比 (m/z)

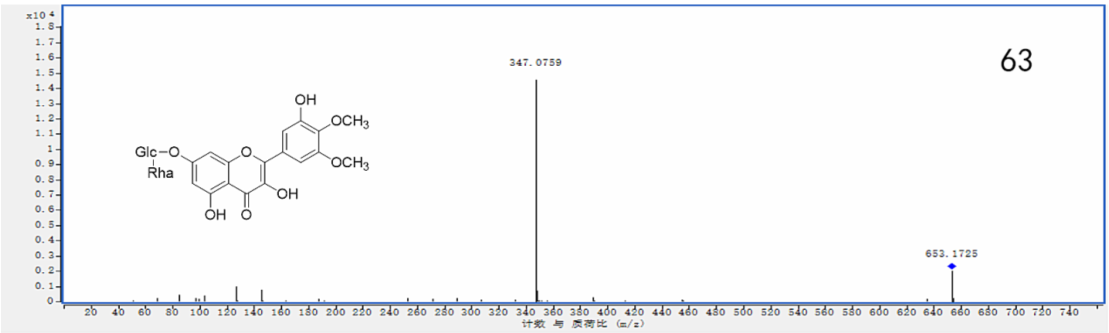
63
347.0759
653.1725
Glc–O
Rha
OH
OCH3
OCH3
OH
OH O
x10 4
计数 与 质荷比 (m/z)

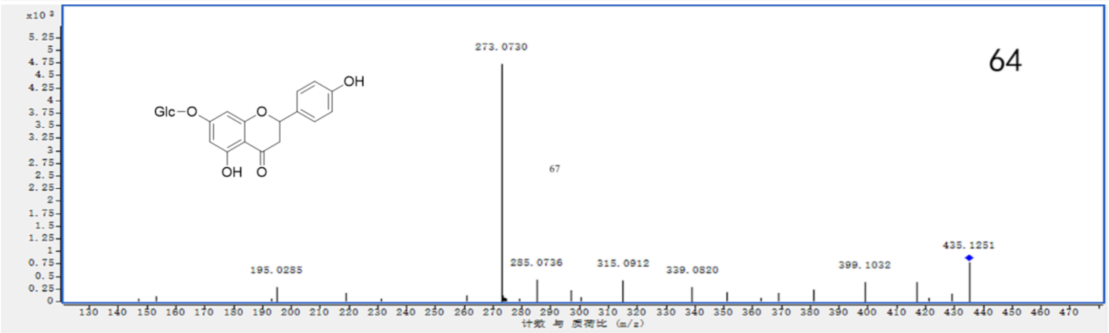
64
273.0730
195.0285
285.0736
315.0912
339.0820
399.1032
435.1251
Glc–O
OH
OH O
x10 3
计数 与 质荷比 (m/z)

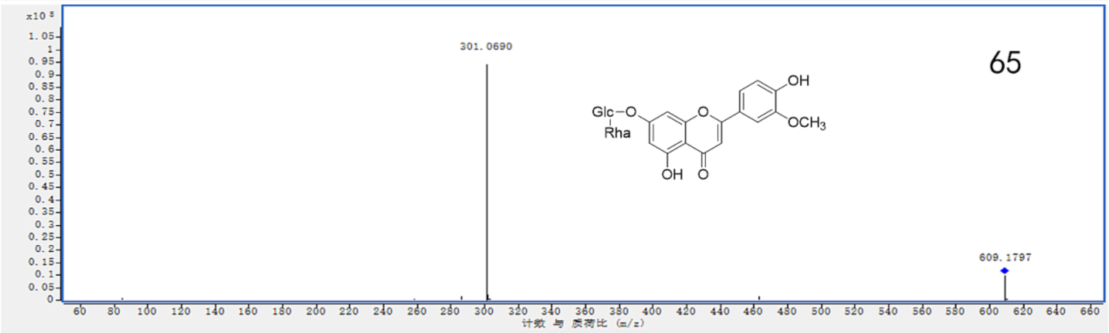
65
301.0690
609.1797
Glc–O
Rha
OH
OCH3
OH O
x10 5
计数 与 质荷比 (m/z)

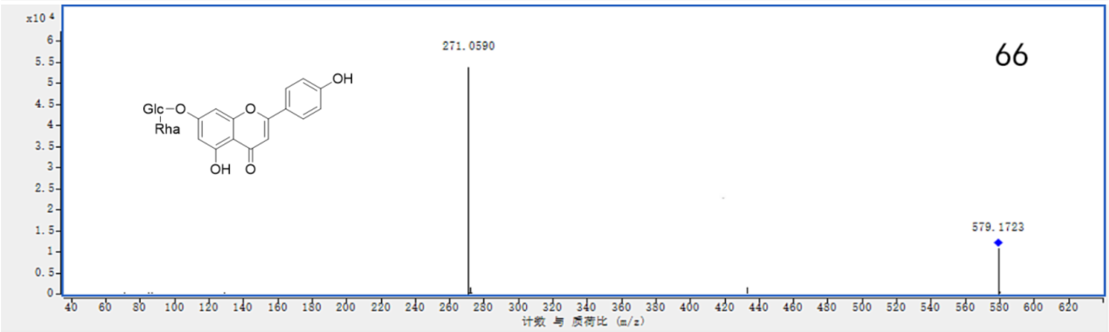
66
271.0590
579.1723
Glc–O
Rha
OH
OH O
x10 4
计数 与 质荷比 (m/z)

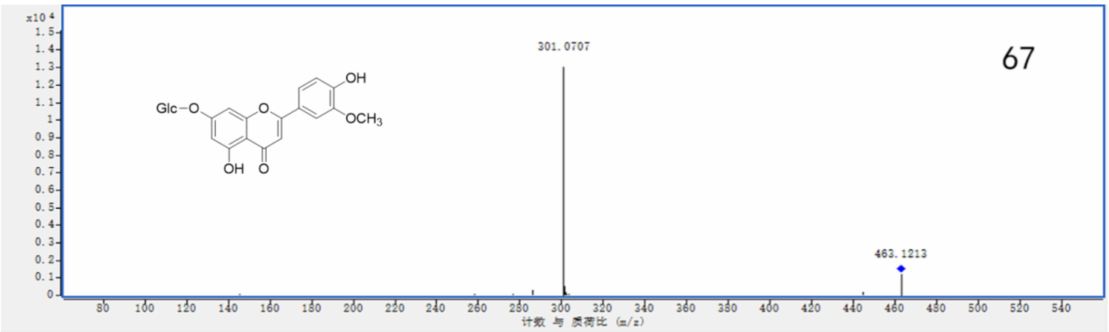
67
301.0707
463.1213
Glc–O
OH
OCH3
OH O
x10 4
计数 与 质荷比 (m/z)

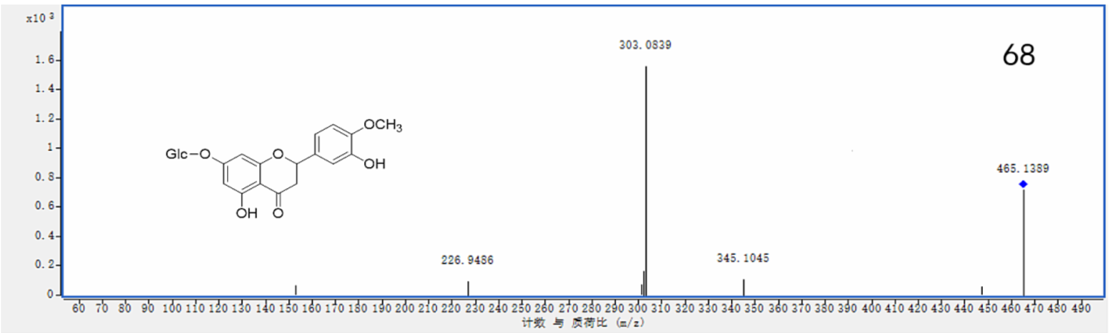
68
x10 3
303.0839
465.1389
226.9486
345.1045
Glc–O
OCH3
OH
OH
O
计数 与 质荷比 (m/z)

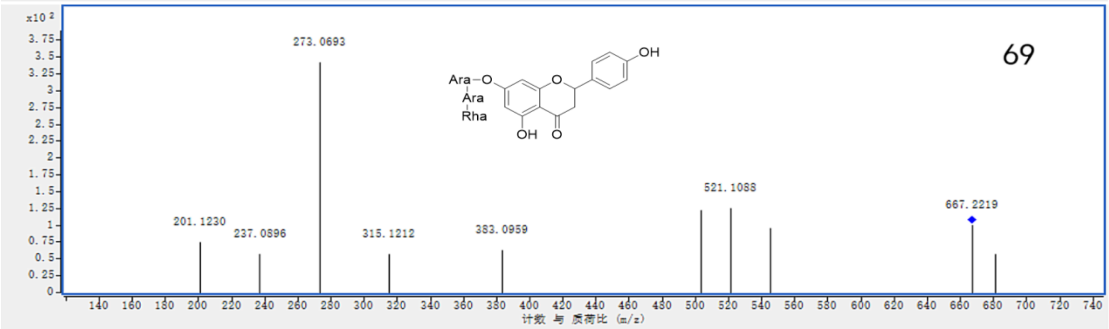
69
x10 2
273.0693
201.1230
237.0896
315.1212
383.0959
521.1088
667.2219
Ara–O
Ara
Rha
OH
OH
O
计数 与 质荷比 (m/z)

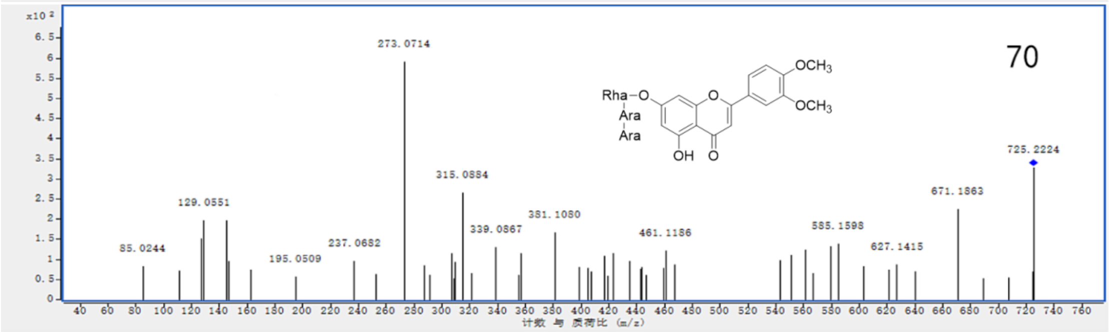
70
x10 2
273.0714
85.0244
129.0551
195.0509
237.0682
315.0884
339.0867
381.1080
461.1186
585.1598
627.1415
671.1863
725.2224
Rha–O
Ara
Ara
OCH3
OCH3
OH
O
计数 与 质荷比 (m/z)

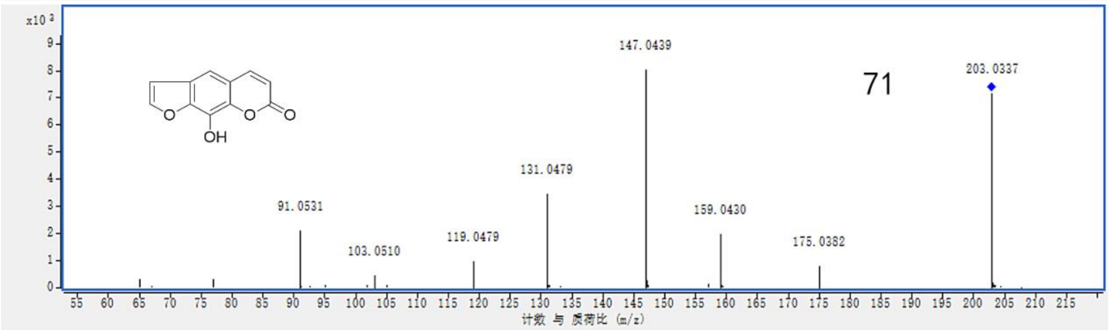
71
x10 3
147.0439
203.0337
91.0531
103.0510
119.0479
131.0479
159.0430
175.0382
OH
O
计数 与 质荷比 (m/z)

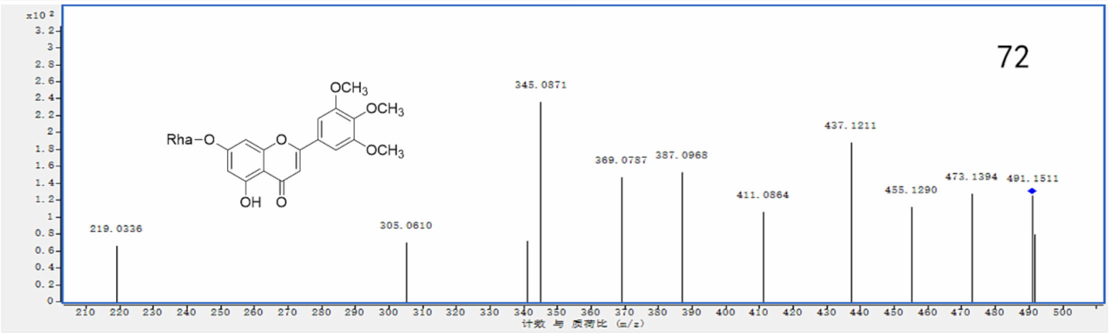
72
x10 2
345.0871
219.0336
305.0610
369.0787
387.0968
411.0864
437.1211
455.1290
473.1394
491.1511
Rha–O
OCH3
OCH3
OCH3
OH
O
计数 与 质荷比 (m/z)

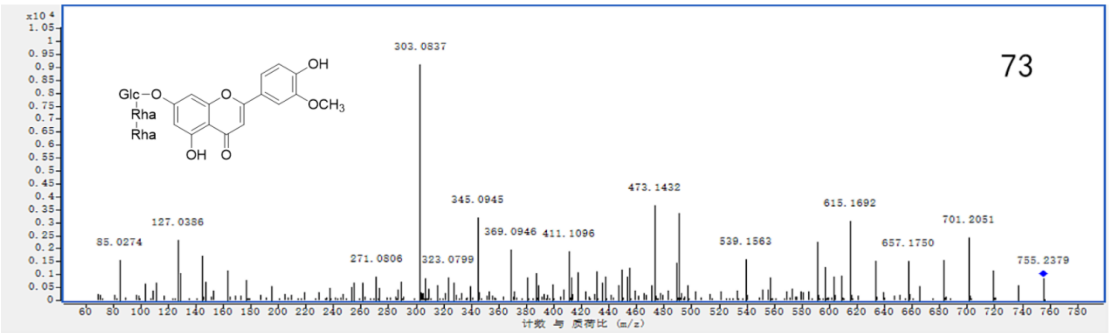

73
x10 4
303. 0837
345. 0945
369. 0946
411. 1096
473. 1432
539. 1563
615. 1692
657. 1750
701. 2051
755. 2379
85. 0274
127. 0386
271. 0806
323. 0799
Glc–O
Rha
Rha
OH
OCH3
OH
O
计数 与 质荷比 (m/z)

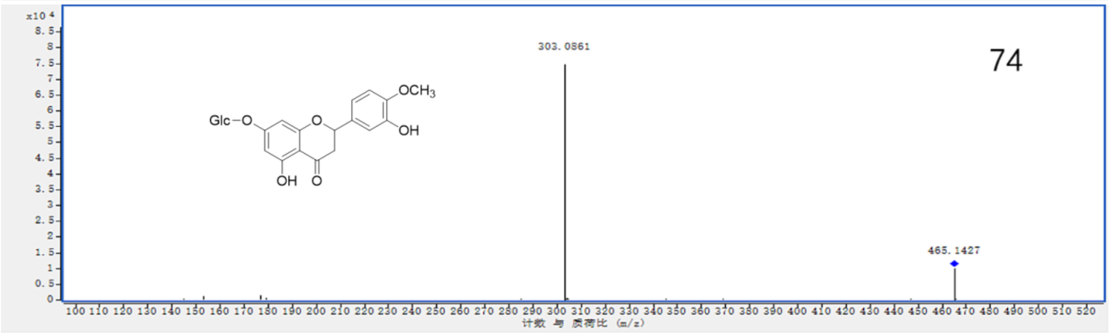

74
x10 4
303. 0861
465. 1427
Glc–O
OCH3
OH
OH
O
计数 与 质荷比 (m/z)

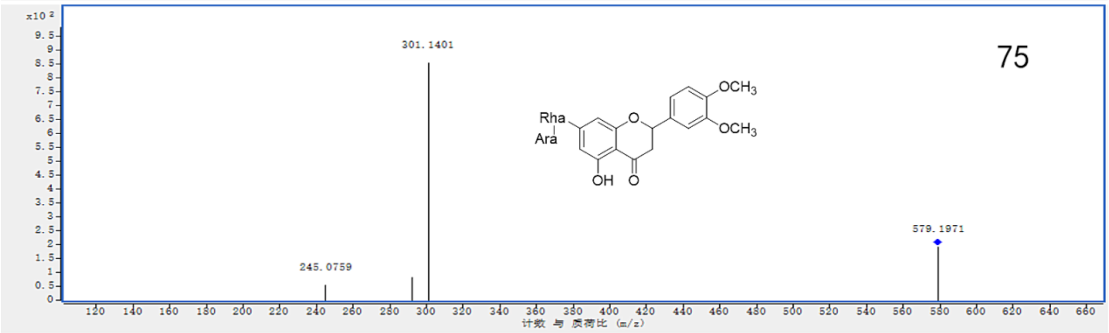

75
x10 2
301. 1401
245. 0759
579. 1971
Rha
Ara
OCH3
OCH3
OH
O
计数 与 质荷比 (m/z)

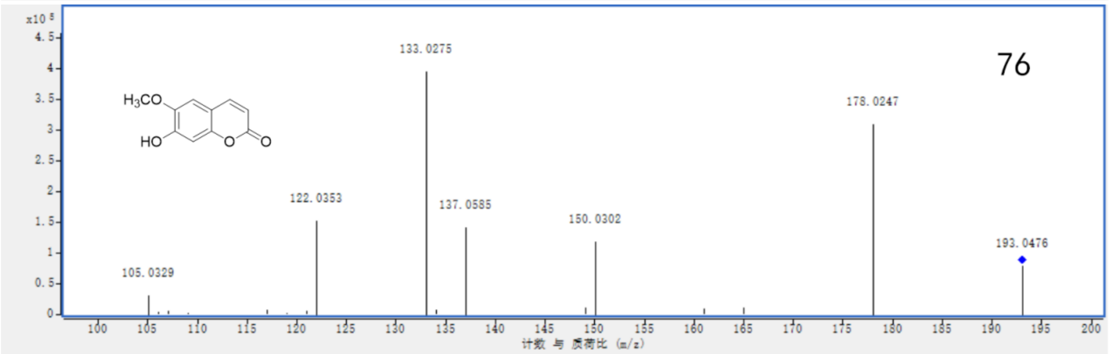

76
x10 5
133. 0275
178. 0247
122. 0353
137. 0585
150. 0302
193. 0476
105. 0329
H3CO
HO
O
计数 与 质荷比 (m/z)

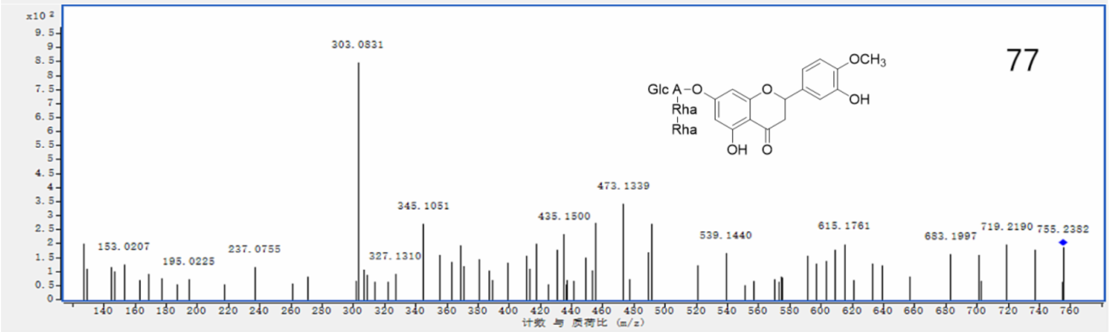

77
x10 2
303. 0831
345. 1051
435. 1500
473. 1339
539. 1440
615. 1761
683. 1997
719. 2190
755. 2382
153. 0207
195. 0225
237. 0755
327. 1310
Glc A–O
Rha
Rha
OCH3
OH
OH
O
计数 与 质荷比 (m/z)

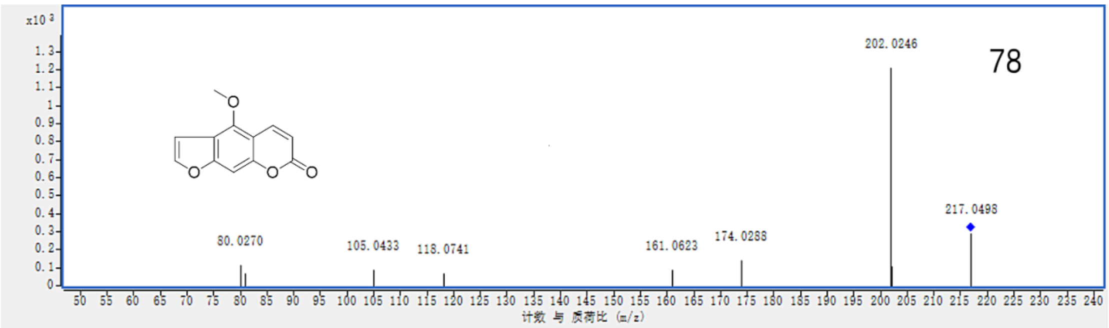
78
x10 3
202.0246
217.0498
80.0270
105.0433
118.0741
161.0623
174.0288
计数 与 质荷比 (m/z)

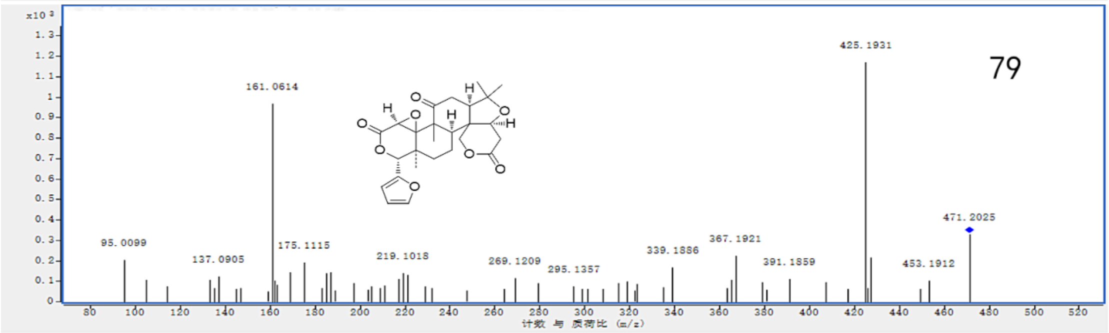
79
x10 3
425.1931
161.0614
471.2025
95.0099
137.0905
175.1115
219.1018
269.1209
295.1357
339.1886
367.1921
391.1859
453.1912
计数 与 质荷比 (m/z)

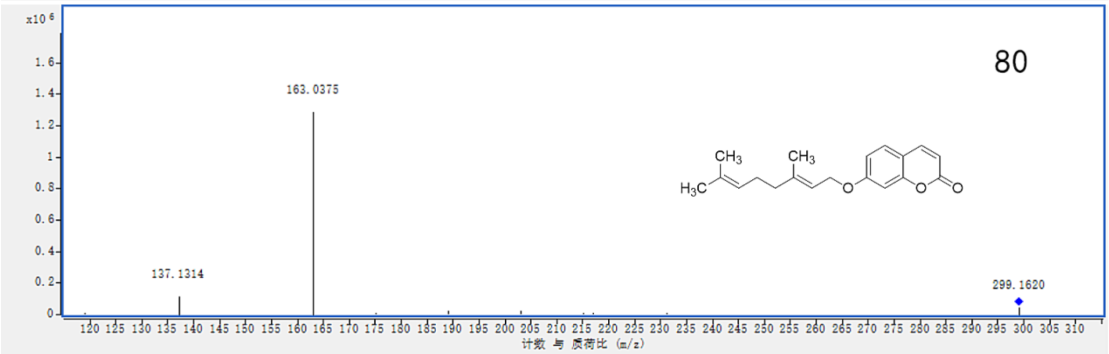
80
x10 6
163.0375
137.1314
299.1620
CH3
CH3
H3C
O
O
O
计数 与 质荷比 (m/z)

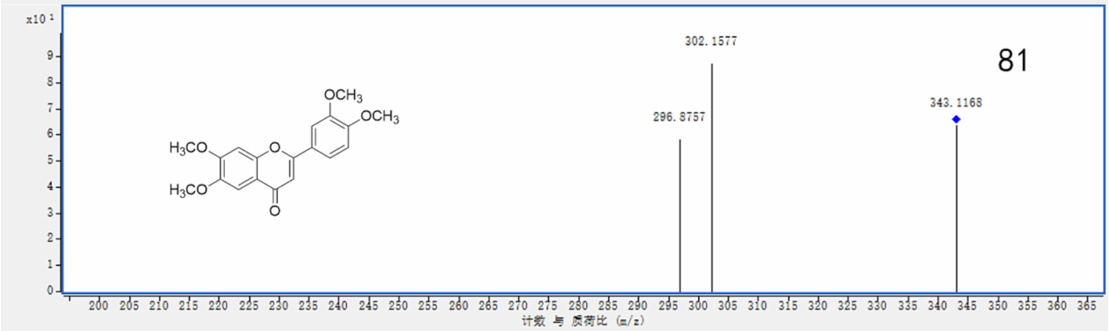
81
x10 1
302.1577
296.8757
343.1168
OCH3
OCH3
H3CO
H3CO
O
O
计数 与 质荷比 (m/z)

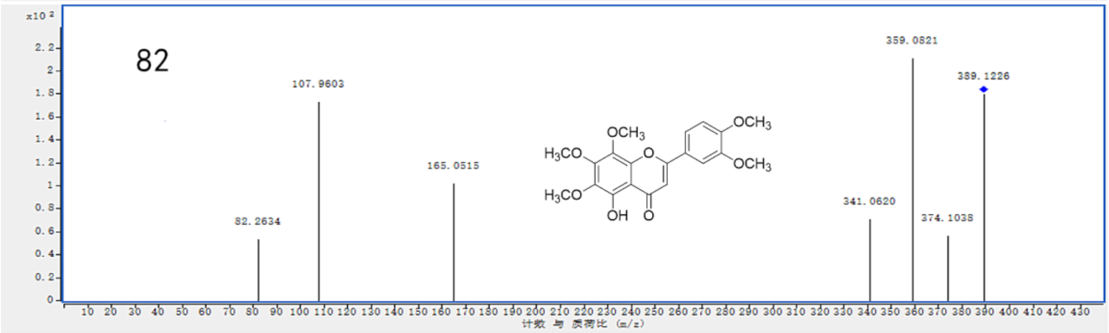
82
x10 2
359.0821
389.1226
107.9603
165.0515
82.2634
341.0620
374.1038
OCH3
OCH3
OCH3
H3CO
H3CO
OH
O
O
计数 与 质荷比 (m/z)

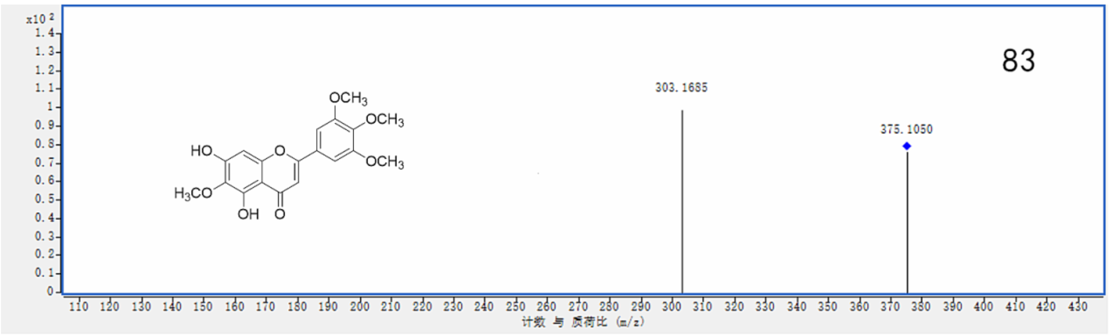
83
303.1685
375.1050
OCH3
OCH3
OCH3
HO
H3CO
OH
O
x10 2
计数 与 质荷比 (m/z)

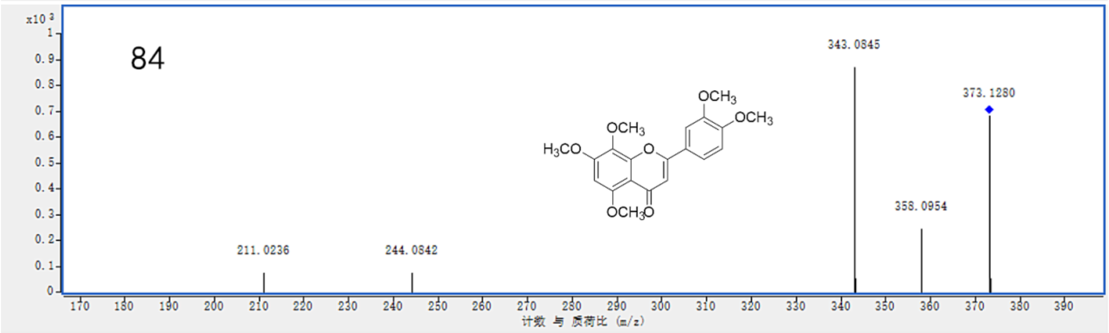
84
343.0845
373.1280
358.0954
211.0236
244.0842
OCH3
OCH3
OCH3
H3CO
OCH3
x10 3
计数 与 质荷比 (m/z)

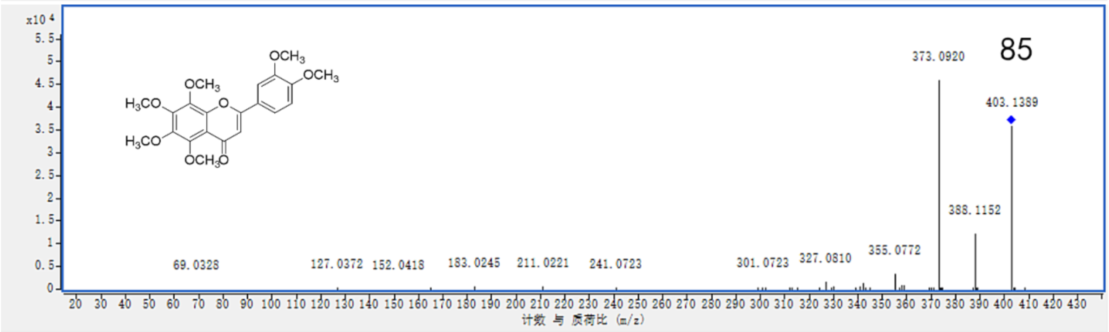
85
373.0920
403.1389
388.1152
69.0328
127.0372
152.0418
183.0245
211.0221
241.0723
301.0723
327.0810
355.0772
OCH3
OCH3
OCH3
H3CO
H3CO
OCH3
x10 4
计数 与 质荷比 (m/z)

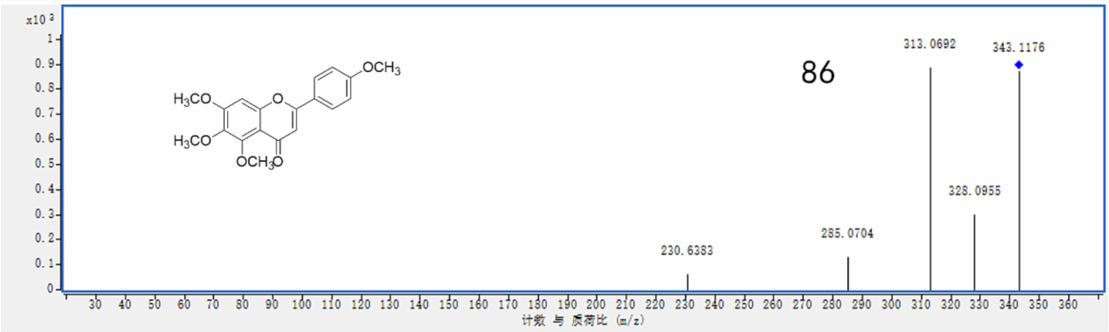
86
313.0692
343.1176
328.0955
285.0704
230.6383
OCH3
H3CO
H3CO
OCH3
x10 3
计数 与 质荷比 (m/z)

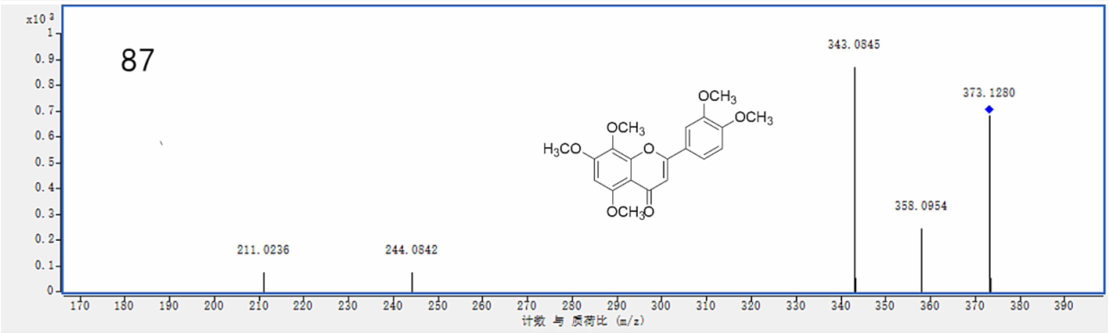
87
343.0845
373.1280
358.0954
211.0236
244.0842
OCH3
OCH3
OCH3
H3CO
OCH3
x10 3
计数 与 质荷比 (m/z)

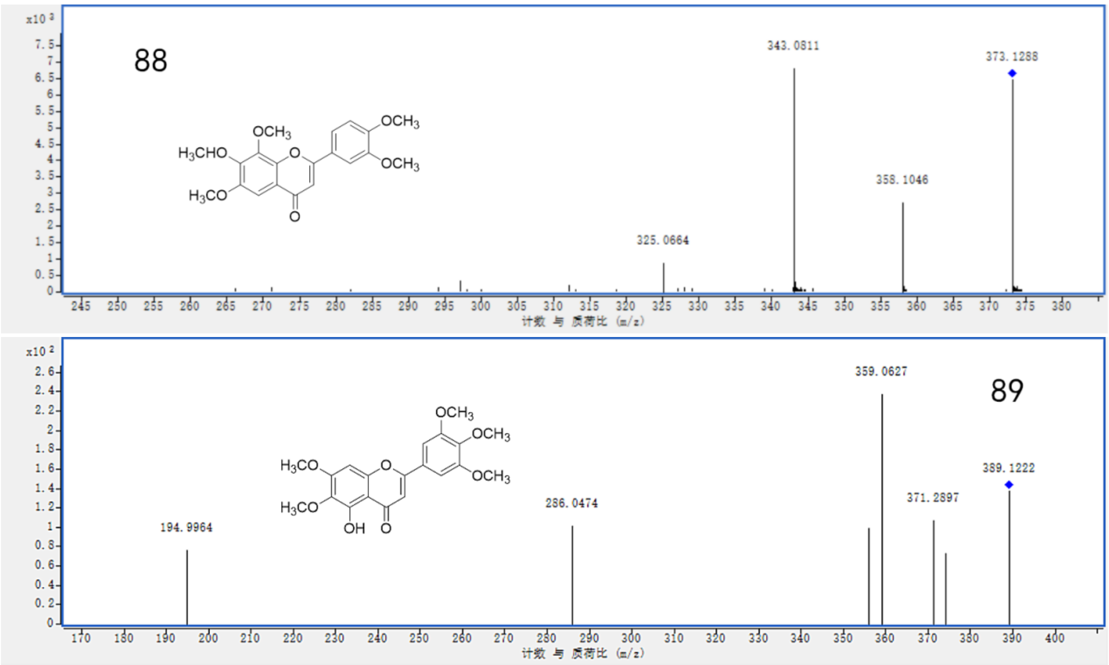

Figure S2. The MS/MS spectra of identified compounds **1**-**89.**
